# Auxin Response Factor 2 (ARF2), ARF3, and ARF4 Mediate Both Lateral Root and Nitrogen Fixing Nodule Development in *Medicago truncatula*

**DOI:** 10.3389/fpls.2021.659061

**Published:** 2021-04-08

**Authors:** Cristina Kirolinko, Karen Hobecker, Jiangqi Wen, Kirankumar S. Mysore, Andreas Niebel, Flavio Antonio Blanco, María Eugenia Zanetti

**Affiliations:** ^1^Instituto de Biotecnología y Biología Molecular, Departamento de Ciencias Biológicas, Facultad de Ciencias Exactas, Universidad Nacional de La Plata, Centro Científico y Tecnológico-La Plata, Consejo Nacional de Investigaciones Científicas y Técnicas, La Plata, Argentina; ^2^Noble Research Institute LLC, Ardmore, OK, United States; ^3^Laboratoire des Interactions Plantes-Microorganismes, INRAE, CNRS, Université de Toulouse, Castanet-Tolosan, France

**Keywords:** auxin response factors, legumes, Nod Factor, miR390, nodulation, root architecture, symbiosis, tasiARFs

## Abstract

Auxin Response Factors (ARFs) constitute a large family of transcription factors that mediate auxin-regulated developmental programs in plants. *ARF2*, *ARF3*, and *ARF4* are post-transcriptionally regulated by the microRNA390 (miR390)/*trans*-acting small interference RNA 3 (*TAS3*) module through the action of *TAS3*-derived *trans****-***acting small interfering RNAs (ta-siRNA). We have previously reported that constitutive activation of the miR390/*TAS3* pathway promotes elongation of lateral roots but impairs nodule organogenesis and infection by rhizobia during the nitrogen-fixing symbiosis established between *Medicago truncatula* and its partner *Sinorhizobium meliloti*. However, the involvement of the targets of the miR390/*TAS3* pathway, i.e., *MtARF2, MtARF3, MtARF4a*, and *MtARF4b*, in root development and establishment of the nitrogen-fixing symbiosis remained unexplored. Here, promoter:reporter fusions showed that expression of both *MtARF3* and *MtARF4a* was associated with lateral root development; however, only the *MtARF4a* promoter was active in developing nodules. In addition, up-regulation of *MtARF2*, *MtARF3*, and *MtARF4a/b* in response to rhizobia depends on Nod Factor perception. We provide evidence that simultaneous knockdown of *MtARF2, MtARF3, MtARF4a*, and *MtARF4b* or mutation in *MtARF4a* impaired nodule formation, and reduced initiation and progression of infection events. Silencing of *MtARF2, MtARF3, MtARF4a*, and *MtARF4b* altered mRNA levels of the early nodulation gene nodulation signaling pathway 2 (*MtNSP2*). In addition, roots with reduced levels of *MtARF2, MtARF3, MtARF4a*, and *MtARF4b*, as well as *arf4a* mutant plants exhibited altered root architecture, causing a reduction in primary and lateral root length, but increasing lateral root density. Taken together, our results suggest that these ARF members are common key players of the morphogenetic programs that control root development and the formation of nitrogen-fixing nodules.

## Introduction

Auxins play essential roles in diverse aspects of plant growth and development, including cell elongation, cell polarity, vascular tissue differentiation, embryo patterning, apical dominance, and leaf shape (Salehin et al., 2015). Auxins also control root system architecture by inhibiting primary root growth and promoting the emergence and growth of lateral roots (Overvoorde et al., 2010). In plants belonging to the nitrogen-fixing clade, which includes the orders Fabales, Fagales, Rosales, and Cucurbitales (Doyle, 2011), these phytohormones play major roles regulating the root nodule symbiosis (Kohlen et al., 2018). Lateral roots serve for soil anchoring as well as water and nutrient acquisition, whereas nodules are the result of a symbiotic relationship with nitrogen-fixing bacteria that allow these plants to overcome nitrogen deficiencies in the soil. It has been proposed that the program of nodule organogenesis, which is activated by bacteria-derived signals known as Nodulation (Nod) factors, has co-opted the endogenous developmental program of root and lateral root development during evolution (Bishopp and Bennett, 2019; Soyano et al., 2021). Up-regulation of meristematic markers such as *WUSCHEL-RELATED HOMEOBOX 5* and *PLETHORA* has been observed during nodule formation (Osipova et al., 2012; Franssen et al., 2015). Notably, two recent reports conducted in *Lotus japonicus* and *Medicago truncatula* provided substantial evidence that the program of lateral root formation has been recruited to contribute to nodule formation in legumes (Schiessl et al., 2019; Soyano et al., 2019). Moreover, although both organs differ in the environmental stimuli and function, their developmental programs converged in the generation and interpretation of auxin maximum (Herrbach et al., 2014; Xiao et al., 2014; Schiessl et al., 2019). In addition, it was recently shown that *Nuclear Factor YA* (*NF-YA*) genes are important regulators of auxin signaling during nodule development *via* the direct control of *SHORT INTERNODES/STYLISH* (*STY*) transcription factor genes and their downstream targets, *YUCCA1* and *YUCCA11*, involved in auxin biosynthesis (Shrestha et al., 2020). This implies that auxin biosynthesis, signaling, and responses must be integrated with the nodulation program, which is initiated by the LysM domain receptor kinases MtNFP (Nod Factor Perception, Amor et al., 2003) and MtLYK3 (Smit et al., 2007) in *M. truncatula* and followed by the activation of a number of transcription factors such as the ERF transcription factor MtERN1 (Ethylene response factor Required for Nodulation 1) (Middleton et al., 2007) and the three subunits of the NF-Y heterotrimeric complex (Combier et al., 2006; Laporte et al., 2014; Baudin et al., 2015). Auxins also interact with the ethylene signaling pathway during root nodule symbiosis, since the ethylene-insensitive *sikle* (*skl*) mutant, which forms numerous infection threads (ITs) and nodules under symbiotic conditions (Penmetsa and Cook, 1997), also exhibited altered auxin transport during nodulation (Prayitno et al., 2006).

Auxin signaling and responses are mediated by multiple members of the Auxin Response Factor (ARF) family of transcription factors and the Aux/IAA proteins. ARF proteins, which have been classified as transcriptional activators or repressors based on sequence analysis and transient expression assays, mediate transcriptional regulation by binding the Auxin response elements (AuxRE) in the promoters of auxin-responsive genes (Ulmasov et al., 1999; Tiwari et al., 2003). At low auxin concentrations, ARF activators form heterodimers with Aux/IAA proteins, which repress auxin-responsive genes by recruiting the TOPLESS repressor. At high auxin concentration, Aux/IAA proteins are ubiquitinated by the SCF E3 ubiquitin ligase complex and targeted to degradation *via* the 26S proteasome, releasing ARF repression and promoting ARF-mediated transcriptional activation of auxin-responsive genes (Guilfoyle and Hagen, 2007).

Depending on the species, plant genomes contain a variable number of ARF members, e.g., 23 ARF members in Arabidopsis, 39 members in *Populus trichocarpa*, 25 members in *Oryza sativa*, 22 members in *Zea mays* (Finet et al., 2013), 24 members in *M. truncatula*, and 51 members in *Glycine max* (Shen et al., 2015). A comprehensive phylogenetic analysis of the ARF family in all major living division of land plants—including eudicots, monocots, gymnosperms, and bryophytes—indicated that ARF genes split into three main clades during evolution: clade A, clade B, and clade C (Finet et al., 2013). In Arabidopsis, ARF activators were clustered mainly in clade A (including AtARF5, AtARF6, AtARF7, and AtARF8), whereas most ARF repressors were divided into both clades B (including AtARF1, AtARF2, AtARF3, AtARF4, and AtARF9) and C (including AtARF10, AtARF16, and AtARF17) (Finet et al., 2013). Several members of the ARF family have been implicated in distinct steps of the formation of lateral roots. In Arabidopsis, single mutants in *AtARF7* or *AtARF19* showed a mild reduction in lateral root number, whereas double *arf7/arf19* mutants exhibited a marked reduction in the development of lateral root primordia, suggesting that these two members of the ARF family might display a certain degree of redundancy in lateral root initiation (Okushima et al., 2005; Wilmoth et al., 2005). *AtARF5* was also implicated in the development of lateral roots. Loss-of-function *arf5* mutants displayed a substantial reduction in the number of emerged lateral roots but exhibited clustering of lateral root primordia, leading to the suggestion that lateral root formation is subjected to a bimodular auxin response control: a first module composed by AtARF7/AtARF19 controls lateral root initiation and a second module involving AtARF5 is required for proper organogenesis of lateral roots (De Smet et al., 2010). Genetic evidence revealed that other ARF members could function as negative regulators of lateral root development. For example, Arabidopsis *arf10/arf16* double mutants produced an increased number of lateral roots (Wang et al., 2005). Interestingly, ARF10, ARF16, and ARF17 have also been involved in the development of nitrogen-fixing nodules and the infection by rhizobia in legumes. Overexpression of the microRNA160, which targets *ARF10*, *ARF16*, and *ARF17* transcripts, leads to a reduction in the number of nodules in *G. max* and *M. truncatula* roots (Bustos-Sanmamed et al., 2013; Turner et al., 2013; Nizampatnam et al., 2015). In addition, three *M. truncatula arf16a-Tnt1* insertional mutants exhibited a reduced number of infection events upon inoculation with its symbiotic partner *Sinorhizobium meliloti* (Breakspear et al., 2014).

*ARF2*, *ARF3*, and *ARF4* are post-transcriptionally regulated by the action of the *trans-*acting small interference RNAs (tasiRNAs)—referred to as tasiARFs—derived from the evolutionarily conserved pathway involving the microRNA390 (miR390) and the *trans*-acting small interference RNA 3 (*TAS3*) transcript (Xia et al., 2017). These members of the ARF family were also implicated in lateral root development in mono- and dicotyledonous species. In Arabidopsis, *arf2*, *arf3*, and *arf4* single mutant plants or plants expressing an artificial microRNA that simultaneously knock down *AtARF2*, *AtARF3*, and *AtARF4* exhibited longer lateral roots and lower lateral root density (Marin et al., 2010; Yoon et al., 2010). Congruently, plants that overproduce tasiARFs—by either activation tagging or overexpression of the *TAS3* gene—also produced longer lateral roots. In *P. trichocarpa*, overexpression of a tasiARF-resistant form of *PtARF4* suppressed lateral root elongation and reduced lateral root density, whereas knockdown of *PtARF4* enhances both lateral root growth and density under normal and salt stress conditions (He et al., 2018). Conversely, Lu et al. (2018) showed that overexpression of *TAS3* in *O. sativa*, which efficiently reduced *OsARF3* mRNA levels, increased the number of lateral roots. These studies indicated that, albeit species-specific differences, the miR390/*TAS3* pathway and its targets *ARF2*, *ARF3*, and *ARF4* play essential roles in the development of lateral roots.

In a previous work, we have shown that the miR390/*TAS3* pathway also mediates the development of nitrogen-fixing nodules (Hobecker et al., 2017). Activation of this pathway by overexpression of miR390b in *M. truncatula* roots led to enhanced lateral root growth, but impaired nodule organogenesis and reduced infection by the nitrogen-fixing bacteria *S. meliloti*. Conversely, inactivation of the miR390/*TAS3* pathway by either expression of a target mimic of miR390 or mutation in the gene encoding ARGONAUTE7—the argonaute protein that binds miR390—increased the density of infection events and the number of nodules and altered their spatial distribution (Hobecker et al., 2017). Thus, the miR390/*TAS3* module functions as a positive modulator of lateral root development and a negative modulator of nodulation in *M. truncatula*. However, whether this pathway operates through their target transcripts *MtARF2, MtARF3*, *MtARF4a*, and *MtARF4b* and the implications of these transcription factors in the development of lateral root organs have not been investigated in legumes. Here, we used *M. truncatula* roots with simultaneous knockdown of *MtARF2, MtARF3*, *MtARF4a*, and *MtARF4b*, as well as an *arf4a Tnt1* insertional mutant, to show that these members of the ARF family contribute to modulation of root architecture and the development of nitrogen-fixing nodules.

## Materials and Methods

### Biological Material

Wild-type (WT) *M. truncatula* Jemalong A17 seeds were obtained from INRA Montpellier, France^1^. *M. truncatula arf4a Tnt-1* insertional mutant seeds were obtained from the Noble Research Institute LLC. *nfp, nf-ya1*, *ern1*, and *skl* mutants were previously described (Amor et al., 2003; Middleton et al., 2007; Laporte et al., 2014; and Penmetsa and Cook, 1997, respectively). *S. meliloti* strain 1021 (Meade and Signer, 1977) or the same strain expressing RFP (Tian et al., 2012) were used for root inoculation as in Hobecker et al. (2017). *Agrobacterium rhizogenes* strain Arqua1 was used for hairy root transformations (Quandt et al., 1993).

### Constructs for Plant Transformation

The *pMtARF3*:*GUS-GFP* construct was generated by amplifying the 1,958-bp region upstream of the translational initiation codon of *MtARF3* using the pMtARF3 F and pMtARF3 R primers listed in Supplementary Table 1. The resulting DNA fragment was cloned into the pENTR/D-TOPO vector (Thermo Scientific) and then recombined into the Gateway-compatible binary vector pKGWFS7,0 (Karimi et al., 2002) using LR Clonase according to the manufacturer’s instructions (Thermo Scientific). The *pMtARF4:GUS-GFP* construct was previously generated by Hobecker et al. (2017). The *ARF2/3/4a/4b* RNAi construct was generated by PCR amplification of an *MtARF3* fragment that contains the binding sites for tasiARFs. PCR was conducted using cDNA from *M. truncatula* roots as template, the *MtARF2/3/4* RNAi F and *MtARF2/3/4* RNAi R primers (Supplementary Table 1) and *pfu* DNA polymerase (Promega). The *GUS* RNAi construct was generated by amplification of a β*-glucuronidase* (*GUS*) fragment using the pKGWFS7,0 vector (Karimi et al., 2002) as template and *GUS* RNAi F and *GUS* RNAi R primers listed in Supplementary Table 1. The *GUS* RNAi and *ARF2/3/4a/4b* RNAi amplified fragments were cloned into the pENTR/D-TOPO (Thermo Scientific) vector and then recombined into the destination vector pK7GWIWG2D (II) (Karimi et al., 2007) to produce *GUS* RNAi and *ARF2/3/4a/4b* RNAi constructs, respectively, following manufacturer’s instructions (Thermo Fisher). The pK7GWIWG2D (II) destination vector contains the *rolD*:*gfp* gene for the detection and selection of transgenic hairy roots; therefore, only roots with detectable GFP fluorescence (more than 80% of the roots) were taken into account for expression and phenotypic analyses. All constructs were verified by sequencing. Binary vectors were introduced into *A. rhizogenes* Arqua1 (Quandt et al., 1993) by electroporation.

### Growth of *Medicago truncatula*, Hairy Root Transformation, Inoculation With Rhizobia and NF Treatment

Seeds were surface sterilized and germinated on 10% (w/v) agar plates at 25°C in the dark for 24 h. Transgenic roots were generated by *A. rhizogenes-*mediated transformation essentially as previously described (Boisson-Dernier et al., 2001) and transferred to Petri dishes containing agar Fahraeus media (Fahraeus, 1957) supplemented with 8 mM KNO_3_ and 12.5 μg/ml of kanamycin for 7 days. Seedlings were grown at 25°C in a 16/8-h day/night cycle with radiation of 200 μmol m^–2^ s^–1^ using mixed lighting containing four OSRAM cool daylight L36W/765 tubes per one OSRAM FLUORA L36W/77 tube. For root architecture analysis, composite plants, consisting of a non-transgenic shoot and transgenic hairy roots, were transferred to slanted boxes containing Fahraeus media supplemented with 8 mM KNO_3_ and grown under the conditions described above for 15 days. For inoculation with rhizobia, plants that developed hairy roots were transferred to slanted boxes containing Fahraeus media free of nitrogen covered with sterile filter paper. Germinated seedlings of WT and the *arf4a* mutant were transferred to Petri dishes containing agar Fahraeus media free of nitrogen for rhizobia inoculation or to the same media supplemented with 8 mM KNO_3_ and grown for 7 or 15 days for root and shoot developmental phenotypic analysis. For rhizobia inoculation, 7 days after transplantation to slanted boxes, seedlings were inoculated with 10 ml of a 1:1,000 dilution of *S. meliloti* 1021 (Meade and Signer, 1977) or the same strain expressing RFP culture grown in liquid TY media until OD_600_ reached 0.8 or with 10 ml of water as a control (mock treatment). The excess of liquid was removed 1 h after inoculation, and seedlings were incubated vertically under the growth conditions described above. For Nod Factor treatment, WT, *nfp, nf-ya1, ern1*, and *skl* mutants were grown on slanted boxes containing Fahraeus media free of nitrogen for 7 days and then treated with 10 ml of a suspension of 10^–8^ M of NFs purified from *S. meliloti* or with 10 ml of water as a mock-treatment.

### Tissue Expression Analysis Using Promoter:Reporter Fusions

Composite plants transformed with the *pMtARF3:GFP-GUS* or *pMtARF4a:GFP-GUS* were transferred to square petri dishes (12 cm × 12 cm) containing slanted agar-Fahraeus medium. GFP fluorescence of roots was visualized with an inverted microscope (Olympus IX51) using UV light with appropriate filters for GFP. For detection of GFP and RFP fluorescence in *S. meliloti* inoculated roots and in nodules, confocal microscopy was performed at 5 and 9 dpi with an *S. meliloti* strain expressing RFP (Tian et al., 2012) using an inverted SP5 confocal microscope (Leica Microsystems). GFP and RFP were excited using 488- and 543-nm lasers, and emissions were collected from 498 to 552 nm and from 578 to 626 nm, respectively. Images were processed with the LAS Image Analysis software (Leica Microsystems).

### Phenotypic Analyses

For analysis of root architecture, germinated WT and *arf4a* mutant seedings, as well as composite *GUS* RNAi and *ARF2/3/4a/4b* RNAi plants generated by *A. rhizogenes-*mediated transformation, were transferred to slanted boxes containing agar-Fahraeus medium supplemented with 8 mM KNO_3_. The number of lateral roots per centimeter of primary root and the length of primary and lateral roots were determined at 7 and 15 days after germination (dag) for WT and *arf4a* mutant plants. The length of aerial part and the number of true leaves of WT and *arf4a* mutants were also determined at 7 and 15 dag. For *GUS* RNAi and *ARF2/3/4a/4b RNAi* composite plants, each root that emerged directly from the sectioned site of the non-transgenic root inoculated with *A. rhizogenes* was considered an independent transgenic primary root in the hairy root system. Only first-order lateral roots that emerged from each independent transgenic primary root were considered to estimate lateral root length and density. The number of lateral roots per centimeter of transgenic primary root and the length of transgenic primary and lateral roots were determined at 15 days after transplantation. For the determination of shoot dry weight, the aerial part of composite plants was dried at 80°C for 24 h and weighed using an analytical balance. Three independent biological replicates were performed. For nodulation analysis, WT, *arf4a* mutants, or composite plants transformed with the *GUS* RNAi or *ARF2/3/4a/4b* RNAi construct were transferred to slanted boxes containing nitrogen-free Fahraeus medium and inoculated with *S. meliloti* 1021 7 days after transplantation. The number of nodules was recorded at different time points after inoculation with *S. meliloti* as previously described (Hobecker et al., 2017). Nodules were classified as pink (nitrogen-fixing mature nodules) or white (immature nodules) according to Traubenik et al. (2020). Only roots containing nodules were considered for the quantification and classification of pink and white nodules. Shoot dry weight and shoot length were determined at 21 dpi with *S. meliloti*. Three independent biological replicates were performed. For analysis of infection events, *GUS* and *ARF2/3/4a/4b* RNAi plants of 14 days after transformation or WT and *arf4a* plants of 14 dag were transferred to petri dishes containing agar-Fahraeus medium free of nitrogen. Seven days after transplantation, roots were inoculated with the *S. meliloti* strain expressing RFP (Tian et al., 2012) and grown as described above. Infection events were visualized, quantified, and classified at 7 dpi in an Olympus IX51 inverted microscope. Infection events were classified as microcolonies, ITs that end in the root hair, ITs that reached the base of the epidermal root hair, or ITs that reached and ramified in the cortical cells. Three independent biological replicates were performed. In all cases, statistical significance of the differences for each parameter was determined by unpaired two-tailed Student’s *t* tests for each construct or for the WT vs. the *arf4a* mutant line.

### RNA Extraction, RT-PCR, and RT-qPCR

Total RNA extraction was performed with Trizol according to the manufacturer’s instructions (Thermo Fisher). RNA concentration was determined by measuring OD_260_ using a Nanodrop ND-1000 (Nanodrop Technologies) and RNA integrity was evaluated by electrophoresis 1.2% (w/v) agarose gels stained with ethidium bromide. Total RNA was treated with DNase I according to the manufacturer’s instructions (Promega) and subjected to first-strand cDNA synthesis using M-MLV reverse transcriptase (Promega). Expression analysis was performed by RT-qPCR using the iQ SBR Green Supermix kit (BioRad) and the CFX96 qPCR system (BioRad) as previously described (Blanco et al., 2009). For each pair of primers, the presence of a unique product of the expected size was verified on 1.2% (w/v) agarose gels stained with ethidium bromide. In all cases, negative controls without template or without RT were included. Expression values were normalized to *MtHIS3L*, which has been validated by GNORM software (Vandesompele et al., 2002), as reported previously by Ariel et al. (2010) and Reynoso et al. (2013). Primers used for RT-qPCR are listed in Supplementary Table 1. For detection of *MtARF4a* mRNAs by semiquantitative RT-PCR on WT and the *arf4a* mutant roots, a pair of primers specific for *MtARF4a* (MtARF4a F and MtARF4a R) were used, which are listed in Supplementary Table 1.

### Sequence Alignment and Phylogenetic Analysis

All members of the Arabidopsis ARF family were retrieved from the TAIR database^2^. *M. truncatula* ARF members were retrieved from the recently released version of the *M. truncatula* genome MtrunA17r5.0-ANR^3^ (Pecrix et al., 2018). Amino acid and nucleotide alignments were generated with the Clustal Omega algorithm available at EMBL-EBI^4^ and decorated with BOXSHADE^5^. The phylogenetic tree was generated with MEGA X (Kumar et al., 2018) using the Neighbor-Joining method (Saitou and Nei, 1987). The percentage of replicate trees in which the associated taxa clustered together in the bootstrap test (10,000 replicates) was computed as described by Felsenstein (1985). The evolutionary distances were computed using the p-distance method (Nei and Kumar, 2000).

### Accession Numbers

Sequence data from this article can be found at the *M. truncatula* genome Mt4.0v1 or MtrunA17r5.0-ANR databases under the following accession numbers, respectively: *MtARF2* (Medtr8g100050 or MtrunA17_Chr8g0385791), *MtARF3* (Medtr2g014770 or MtrunA17_Chr2g0282961), *MtARF4a* (Medtr4g060460 or MtrunA17_Chr4g0029671), *MtARF4b* (Medtr2g093740 or MtrunA17_Chr2g0326281), *MtARF16a* (Medtr1g094960 or MtrunA17_Chr1g0199681), *MtARF19a* (Medtr2g018690 or MtrunA17_Chr2g0301251), *MtNSP1* (Medtr8g020840 or MtrunA17_Chr8g0344101), *M*t*NSP2* (Medtr3g072710 or MtrunA17_Chr3g0114841), *MtNIN* (Medtr5g099060 or MtrunA17_Chr5g0448621), *MtERN1* (Medtr7g085810 or MtrunA17_Chr7g0253424), *MtNF-YA1* (MtrunA17_Chr1g0148951), *MtENOD40* (MtrunA17_Chr8g0368441), *MtNFP* (MtrunA17_Chr5g0403371), and *MtSKL* (MtrunA17_Chr5g0427621).

## Results

### *MtARF3* and *MtARF4a* Are Expressed During Lateral Root Development, but Only *ARF4a* Is Transcribed During the Root Nodule Symbiosis

**FIGURE 1:**
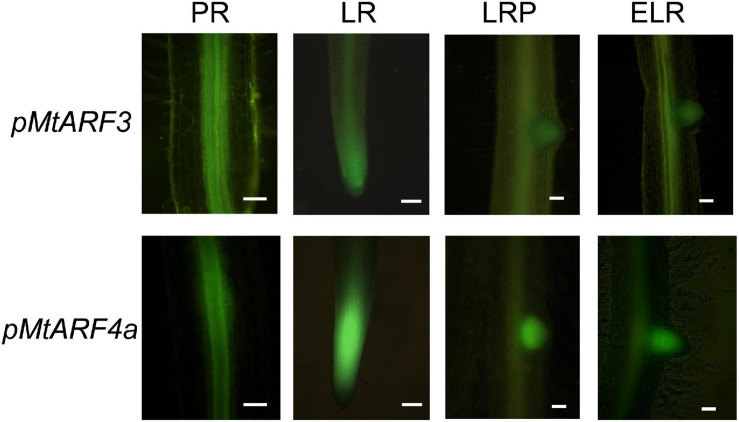
Promoter:reporter fusion expression analysis of *MtARF3* and *MtARF4a* in primary and lateral roots. Expression of the *GFP* reporter gene in roots transformed with the *pMtARF3:GUS-GFP* (upper panels) or the p*MtARF4a:GUS-GFP* (bottom panels) construct was detected in the vasculature of the primary root (PR) and lateral root (LR), in lateral root primordia (LRP) prior to emergence and in emerged lateral roots (ELR). Bars = 50 μm.

**FIGURE 2:**
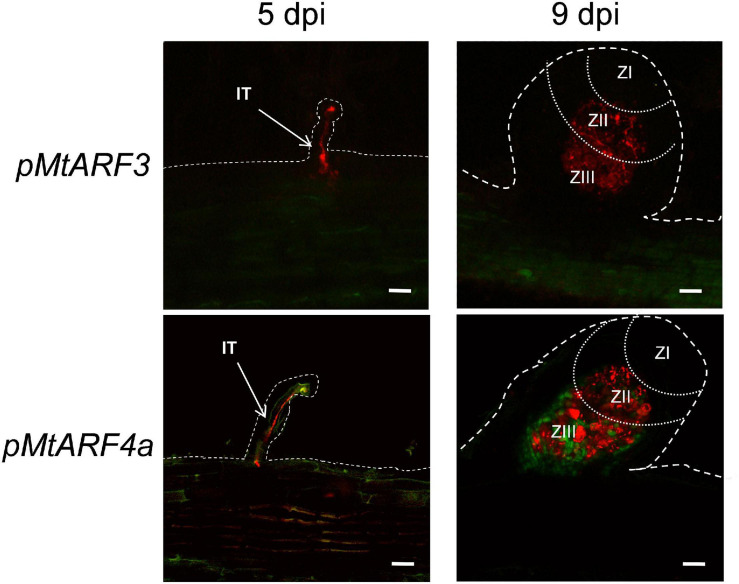
Promoter:reporter fusion expression analysis of *MtARF3* and *MtARF4a* at different stages of the symbiotic interaction with *S. meliloti.* GFP fluorescence was analyzed in roots transformed with the *pMtARF3:GUS-GFP* (upper panels) or the *pMtARF4a:GUS-GFP* (bottom panels) construct at 5 days post-inoculation (dpi) and in developing nodules at 9 dpi with a strain of *S. meliloti* that expressed the red fluorescent protein (RFP). Dashed lines mark the epidermal cells of roots and nodules. Dotted lines separate different nodule zones. ZI, meristematic zone; ZII, infection zone; ZIII, fixation zone; IT, infection thread. Bars = 50 μm.

Considering that expression of the *MIR390* promoter was associated with the development of lateral roots and nodules, we aimed to characterize the spatial and temporal expression pattern of the targets of the miR390/*TAS3* pathway during the development of both types of lateral organs. Previous phylogenetic analysis of the ARF family using ESTs or the Mt3.5 version of the *M. truncatula* genome identified single genes as putative orthologs of Arabidopsis *AtARF2* and *AtARF3*, referred to as *MtARF2* and *MtARF3*, respectively, and two genes evolutionarily closer to Arabidopsis *AtARF4*, designated as *MtARF4a* and *MtARF4b* (Zhou et al., 2013; Shen et al., 2015). Proteins encoded by *MtARF4a* and *MtARF4b* exhibited 75% of identity to each other (Supplementary Figure 1). Here, we generated a phylogenetic tree that includes all members of the Arabidopsis ARF family, and MtARF2, MtARF3, MtARF4a, and MtARF4b. In addition, the tree includes *M. truncatula* ARF members that have been involved in root development and nodulation in this legume, i.e., MtARF10, MtARF16, and MtARF17 (Bustos-Sanmamed et al., 2013; Breakspear et al., 2014), as well as best *M. truncatula* homologs of ARF members implicated in these processes in other plant species, such as AtARF5, AtARF7, and AtARF19 in lateral root development (Okushima et al., 2005; Wilmoth et al., 2005; De Smet et al., 2010) and GmARF6 and GmARF8 in nodulation (Wang et al., 2015). The amino acid sequences of *M. truncatula* ARF members were retrieved from the recently released version of the *M. truncatula* genome MtrunA17r5.0-ANR (Pecrix et al., 2018). This phylogenetic analysis verified that MtARF2, MtARF3, MtARF4a, and MtARF4b clustered in the same clades as their Arabidopsis counterparts (Supplementary Figure 2). Inspection of public RNA-sequencing data reported by Schiessl et al. (2019) indicated that *MtARF4a* increased at 5 days after spot-inoculation with droplets of a *S. meliloti* suspension, whereas *MtARF4b* decreased at all time points after inoculation (Supplementary Figure 3). On the other hand, *MtARF3* transcript levels significantly increased in fully emerged lateral roots (72 h after induction of lateral root formation) but decreased at 7 days after spot-inoculation with rhizobia, whereas *ARF2* levels did not increase neither during lateral root development nor after spot-inoculation with rhizobia (Supplementary Figure 3). To better characterize the spatial expression pattern of *AR*F genes during lateral root and nodule formation promoter:*GUS-GFP* transcriptional fusions were generated for *MtARF3* and *MtARF4a* (*pMtARF3:GFP-GUS* and *pMtARF4:GFP-GUS*, respectively). Despite several attempts using different combinations of primers based on either the *M. truncatula* genome v4.0 or v5.0, we were unable to amplify a promoter region of *ARF2*. Introduction of *pMtARF3:GFP-GUS* and *pMtARF4a:GFP-GUS* constructs into *M. truncatula* hairy roots revealed that both promoters were active in the vascular tissue of the primary roots, as well as in the vasculature and the meristematic region of lateral roots. *pMtARF3* and *pMtARF4a* expression was also detected in lateral root primordia prior to emergence and in emerged lateral roots (Figure 1). To evaluate the expression of *pARF3* and *pARF4* during symbiosis, hairy roots harboring *pMtARF3:GFP-GUS* or *pMtARF4a:GFP-GUS* were inoculated with a *S. meliloti* strain that expresses the red fluorescent protein (RFP) (Tian et al., 2012). This strain allows visualizing the root hairs that contain an IT, i.e., the tubular structure that allows the bacteria to cross the epidermis and reach the dividing cells of the nodule primordia, as well as infected cells of the nodule. Upon rhizobia inoculation, GFP fluorescence was not detected in the infected root hair or in the adjacent epidermal cells at 5 dpi, or in the infected or non-infected cells of developing nodules of 9 dpi when roots were transformed with the *pMtARF3:GFP-GUS* construct (Figure 2). On the other hand, expression of GFP driven by *pMtARF4a* promoter was found in the root hair containing elongating ITs and in the epidermal cells surrounding the infected root hair at 5 dpi, as well as in the non-infected cells that surround infected cells of nodules of 9 dpi (Figure 2), in agreement with previous results (Hobecker et al., 2017). Thus, the promoter reporter analysis presented here revealed that the activity of both *pMtARF3* and *pMtARF4a* is associated with lateral root development. However, only *pMtARF4a* seems to be responsive to rhizobial infection and active in developing nodules, consistently with the mRNA expression pattern described in spot-inoculation experiments (Schiessl et al., 2019).

### Up-Regulation of *MtARF2*, *MtARF3*, and *MtARF4a/b* mRNAs Depends on the Nod Factor Signaling Pathway

**FIGURE 3:**
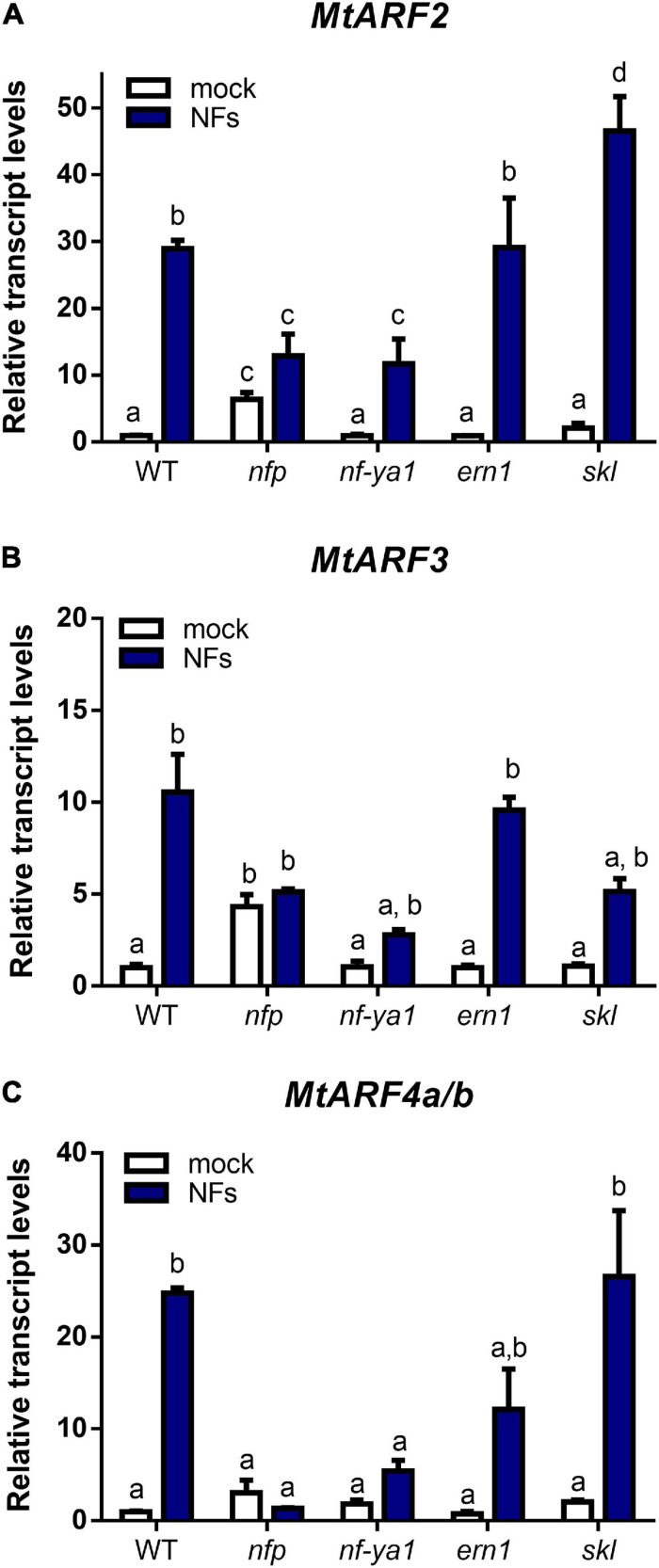
*MtARF2*, *MtARF3*, and *MtARF4a/b* are up-regulated by Nod Factors and depends on *MtNFP* and *MtNF-YA1* genes. Transcript levels of *MtARF2* **(A)**, *MtARF3* **(B)**, and *MtARF4a/b* **(C)** were analyzed in root tissue of WT and *nfp, nf-ya1*, *ern1*, and *skl* mutant plants treated with 10^–8^ M purified Nod Factors (NFs, blue bars) or water (mock, white bars) for 48 h. Expression levels were determined by RT-qPCR and normalized to the levels of the *MtHIS3L* transcript. Values are expressed relative to the WT mock-treated sample, which was set at 1. Different letters above the bars indicate statistically significant differences between samples in an unpaired two-tailed Student’s *t* test with a *p* value ≤ 0.05.

Previously, we have shown that mRNA levels of *MtARF2*, *MtARF4a/b*, and, to a lesser extent, *MtARF3* increased in *M. truncatula* roots upon inoculation with *S. meliloti* (Reynoso et al., 2013; Hobecker et al., 2017). Here, we investigated whether the Nod Factors (NFs) and their signaling pathway were responsible for up-regulation of this set of ARFs during symbiosis. Roots of WT plants were treated with purified NFs from *S. meliloti* for 48 h. Reverse transcription quantitative PCR (RT-qPCR) analysis revealed that these ARFs were extensively up-regulated by NFs; *MtARF2* and *MtARF4a/b* mRNA levels increased by more than 25-fold in NF-treated roots as compared to mock-inoculated WT roots, whereas the increase was 12-fold for *MtARF3* transcripts (Figure 3). Interestingly, mRNA levels of none of these *ARFs* were significantly up-regulated upon NF treatment in the *nfp* mutant, a loss-of-function mutant in the *MtNFP* gene involved in NF reception (Amor et al., 2003), albeit levels of *MtARF2* and *MtARF3* were slightly higher in this mutant than in WT under mock inoculation conditions. In addition, up-regulation of *MtARF2* and *MtARF3* or *MtARF4a/b* in response to NFs was partially or completely impaired, respectively, in mutants of the transcription factor *MtNF-YA1*. Up-regulation of *MtARF2* and *MtARF3* mRNA levels was not impaired in *ern-1* and *skl* mutants (Figures 3A,B). Moreover, *MtARF2* mRNA levels increased to a higher extent in *skl* mutants as compared to WT plants, which might be consistent with the enhanced auxin transport observed in *skl* (Prayitno et al., 2006). On the other hand, up-regulation of *MtARF4a/b* in response to NFs was partially impaired in *ern1* and unaffected in *skl* mutants (Figure 3C). Altogether, these results suggest that induction of these ARFs, notably of *MtARF4a/b*, depends on NF perception and requires the function of the MtNF-YA1 transcription factor.

### Knockdown of *MtARF2, MtARF3, MtARF4a*, and *MtARF4b* and Mutation of *MtARF4a* Reduce Nodulation and Infection by Rhizobia

**FIGURE 4:**
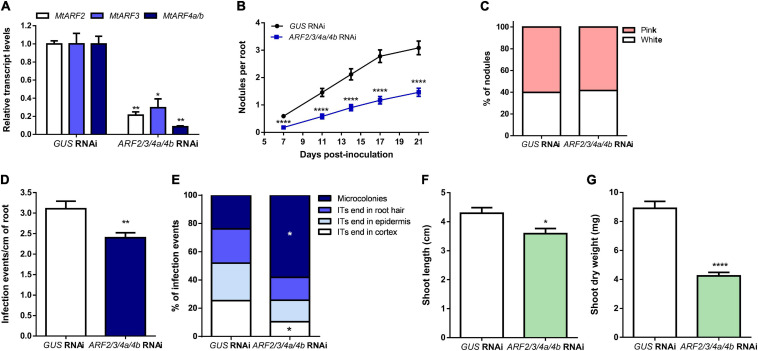
Simultaneous knockdown of *MtARF2, MtARF3, MtARF4a*, and *MtARF4b* impaired nodule formation and infection by rhizobia. **(A)** Expression levels of *MtARF2*, *MtARF3*, and *MtARF4a/b* transcripts in *GUS* RNAi and *ARF2/3/4a/4b* RNAi roots. Expression values were determined by RT-qPCR, normalized to *MtHIS3L*, and expressed relative to the *GUS* RNAi sample, which was set at 1. Each bar represents the mean ± SE of three biological replicates (whole root tissue from at least three composite plants were collected in each biological replicate) with three technical replicates each. Asterisks indicate statistically significant differences between *GUS* and *ARF2/3/4a/4b* RNAi roots in an unpaired two-tailed Student’s *t* test (^∗^*p* ≤ 0.05, ^∗∗^*p* ≤ 0.01). **(B)** Time-course nodule formation in *GUS* and *ARF2/3/4a/4b* RNAi roots upon inoculation with *S. meliloti*. Error bars represent mean ± SE of three independent biological replicates, each with at least 50 roots. Four asterisks indicate statistically significant differences between *GUS* and *ARF2/3/4a/4b* RNAi roots in an unpaired two-tailed Student’s *t* test with a *p* value ≤ 0.0001. **(C)** Percentage of pink and white nodules developed in *GUS* RNAi and *ARF2/3/4a/4b* RNAi roots at 21 dpi. Bars represent the mean ± SE of three independent biological replicates. More than 68 nodules from more than 15 independent plants per construct were quantified in each biological replicate. **(D)** Density of infection events in *GUS* and *ARF2/3/4a/4b* RNAi roots at 7dpi with a *S. meliloti* strain expressing the RFP protein. Each bar represents the mean ± SE of three biological replicates, each with more than 25 transgenic roots. The asterisks indicate statistically significant differences between *GUS* and *ARF2/3/4a/4b* RNAi roots in an unpaired two-tailed Student’s *t* test with a *p* value ≤ 0.01. **(E)** Progression of infection events in *GUS* and *ARF2/3/4a/4b* RNAi roots. Infection events were classified as microcolonies, infection threads (ITs) that end in the root hair, in the epidermal cell layer, or reach the cortex at 7 dpi. Each category is presented as the percentage of total infection events. Data are representative of three independent biological replicates, each with more than 25 transgenic roots. The asterisk indicates that the percentage of microcolonies and ITs that end in cortex was significant different between *GUS* and *ARF2/3/4a/4b* RNAi roots in an unpaired two-tailed Student’s *t* test with a *p* value ≤ 0.05. **(F,G)** Shoot length **(F)** and shoot dry weight **(G)** measured in *GUS* and *ARF2/3/4a/4b* RNAi composite plants grown on free-nitrogen slanted agar-Fahraeus at 21 dpi with *S. meliloti*_._ Error bars represent the mean ± SE of three biological replicates, each performed with more than 10 composite plants. Asterisks denote a statistically significant difference between *GUS* and *ARF2/3/4a/4b* RNAi composite plants in an unpaired two-tailed Student’s *t* test (^∗^*p* ≤ 0.05, ^****^p ≤ 0.0001).

To elucidate whether these three ARF members play a role in the control of nodule formation and/or rhizobial infection in *M. truncatula*, we applied an RNA interference (RNAi) strategy to simultaneously knock down *MtARF2, MtARF3, MtARF4a*, and *MtARF4b*. The fragment used for RNAi was designed in an mRNA region highly conserved across *MtARF2, MtARF3, MtARF4a*, and *MtARF4b* that includes the tasiARFs target sites (Supplementary Figure 4). Introduction of the *ARF2/3/4a/4b* RNAi construct in *M. truncatula* hairy roots reduced by 80, 70, and more than 90% *MtARF2*, *MtARF3*, and *MtARF4a/b* mRNA levels, respectively, relative to the levels of *GUS* RNAi roots used as the control (Figure 4A). However, this RNAi construct did not decrease levels of *MtARF16a* or *MtARF19a* (Supplementary Figure 5), evidencing the specificity of the RNAi approach. Reductions of *MtARF2, MtARF3, MtARF4a*, and *MtARF4b* transcript levels significantly impacted nodulation, reducing the number of nodulated plants, as well as the number of nodules formed over the time in hairy roots (Table 1 and Figure 4B). These differences were observed as early as 7 dpi, with a 70% reduction in the number of nodules formed in *ARF2/3/4a/4b* RNAi as compared with *GUS* RNAi plants, but continued over the time course of the experiment, with nearly a 50% reduction in the number of nodules at 21 dpi. However, the percentage of nodules that acquired the characteristic pink color caused by expression of leghemoglobin upon the onset of nitrogen fixation was not affected by knockdown of *MtARF2, MtARF3, MtARF4a*, and *MtARF4b* (Figure 4C). These results suggest that silencing of these *ARFs* negatively affects nodule formation, but once formed, these nodules develop the nitrogen fixation zone characteristic of indeterminate nodules. To evaluate whether infection by rhizobia was affected by knockdown of *MtARF2, MtARF3, MtARF4a*, and *MtARF4b*, the number of infection events and their progression were evaluated in hairy roots inoculated with the RFP-expressing *S. meliloti* strain. Knockdown of *MtARF2, MtARF3*, and *MtARF4a/b* caused a significant reduction in the total density of infection events, i.e., the number of infection events per centimeter of root (Figure 4D). A more notable effect was observed in the progression of the infection events, with 58% of the infection events remaining at the microcolony stage and only 10% of the ITs reaching the dividing cortical cells in *ARF2/3/4a/4b* RNAi roots, whereas over 50% of the infection events reached the base of the epidermal cells or reached the cortex in *GUS* RNAi roots (Figure 4E). Thus, these results indicate that simultaneous silencing of *MtARF2, MtARF3, MtARF4a*, and *MtARF4b* interferes not only with the initiation of infection events but also with their progression to the dividing cortical cell beneath the infection site. In addition, *ARF2/3/4a/4b* RNAi composite plants grown in the absence of nitrogen exhibited reduced shoot length and shoot dry weight as compared to *GUS* RNAi at 21 dpi with *S. meliloti* (Figures 4F,G, respectively), presumably because of poor nodulation and impaired infection that compromises nitrogen fixation.

**TABLE 1:** Number and percentage of plants with nodules.

Days post-inoculation	*GUS* RNAi	*ARF2/3/4a/4b* RNAi
7	44/108 (40.8%)	21/134 (15.7%)
11	80/108 (74.1%)	45/134 (33.6%)
14	85/108 (78.7%)	56/134 (41.8%)
17	88/108 (81.5%)	74/134 (55.2%)
21	96/108 (88.9%)	84/134 (62.7%)

**FIGURE 5:**
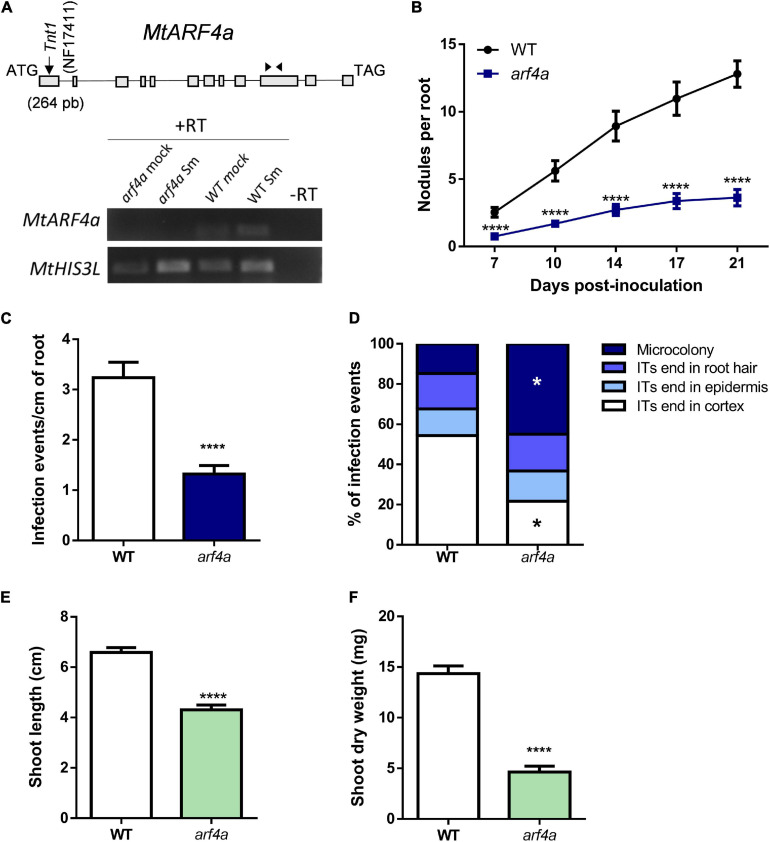
A *Tnt1* insertional mutant in *MtARFa* gene (*arf4a*) exhibited reduced nodulation and infection by rhizobia. **(A)** Schematic representation of *MtARF4a* gene model and the insertion of the *Tnt1* transposon (upper panel). The gene model is composed of 12 exons (gray blocks) and 11 introns (lines). The position of the *Tnt1* insertion within the first exon in the NF17411 mutant line is indicated in base pairs (bp) with a black arrow. Black arrowheads indicate the position of MtARF4a F and MtARF4a R primers specific for *ARF4a* (listed in Supplementary Table 1) used for RT-PCR analysis. Expression levels of *MtARF4a* and *MtHIS3L* were determined by semiquantitative RT-PCR using 35 and 25 cycles, respectively, on WT and NF17411 (*arf4a*) roots at 48 hpi with *S. meliloti* (Sm) or water (mock) (lower panel). **(B)** Time-course nodule formation in roots of WT and *arf4a* plants upon inoculation with *S. meliloti*. Error bars represent the mean ± SE of three biological replicates, each performed with at least ten plants. Four asterisks indicate statistically significant differences between WT and *arf4a* mutant roots in an unpaired two-tailed Student’s *t* test with a *p* value ≤ 0.0001. **(C)** Density of infection events developed at 7 dpi in WT and *arf4a* mutant roots. Error bars represent the mean ± SE of three biological replicates each performed with at least 10 plants. Four asterisks indicate statistically significant differences between WT and *arf4a* mutant roots in an unpaired two-tailed Student’s *t* test with a *p* value ≤ 0.0001. **(D)** Progression of infection events in WT and *arf4a* mutant roots. Infection events were classified as microcolonies or ITs that end in the root hair, in the epidermal cell layer, or reach the cortex at 7 dpi. Each category is presented as the percentage of total infection events. Results are representative of three biological replicates, each with more than 10 plants. The asterisk indicates that the percentage of microcolonies and ITs that end in cortex was significantly different in an unpaired two-tailed Student’s *t* test between WT and *arf4a* roots with *p* ≤ 0.05. **(E,F)** Shoot length **(E)** and shoot dry weight **(F)** measured in WT and *arf4a* mutant plants grown on free-nitrogen slanted agar-Fahraeus at 21 dpi with *S. meliloti*_._ Error bars represent the mean ± SE of three biological replicates, each performed with more than 10 plants. Four asterisks denote a statistically significant difference between WT and *arf4a* plants in an unpaired two-tailed Student’s *t* test with a *p* value ≤ 0.0001.

Since the *MtARF4a* promoter was active during bacterial infection and nodule formation and up-regulation of *ARF4a/b* was completely impaired in *nfp* and *nf-ya1* mutants, we analyzed the symbiotic phenotype caused by a mutation in the *MtARF4a* gene. A mutant carrying the *Tnt1* insertion within the first exon of the *MtARF4a* gene (*arf4a*) was obtained by screening the collection available at Noble Research Institute LLC (Tadege et al., 2008; Cheng et al., 2014). Semiquantitative RT-PCR analysis revealed that levels of *MtARF4a* mRNAs were undetectable in *arf4a* mutants, whereas WT plants accumulated noticeable levels of *MtARF4a* transcripts (Figure 5A). Upon inoculation with rhizobia, the *arf4a* mutant developed a significantly lower number of nodules as compared with WT plants (Figure 5B). In agreement with what was observed in *ARF2/3/4a/4b* RNAi roots, roots of *arf4a* mutants exhibited a significant reduction in the density of the infection events (Figure 5C) as well as in their progression. In the *arf4a* mutants, 45% of the infection events remained at the microcolony stage and only 20% reached the root cortex, whereas in WT roots, nearly 55% of the infection events reached the cortical cells (Figure 5D). Reduced nodule formation and bacterial infection also impacted the development of the aerial part of the *arf4a* plants grown in the absence of nitrogen, which exhibited reduced shoot length and dry weight as compared with WT plants under symbiotic conditions (Figures 5E,F).

### Knockdown of *MtARF2, MtARF3, MtARF4a*, and *MtARF4b* Impairs Expression of Nodulation Signaling Pathway 2

**FIGURE 6:**
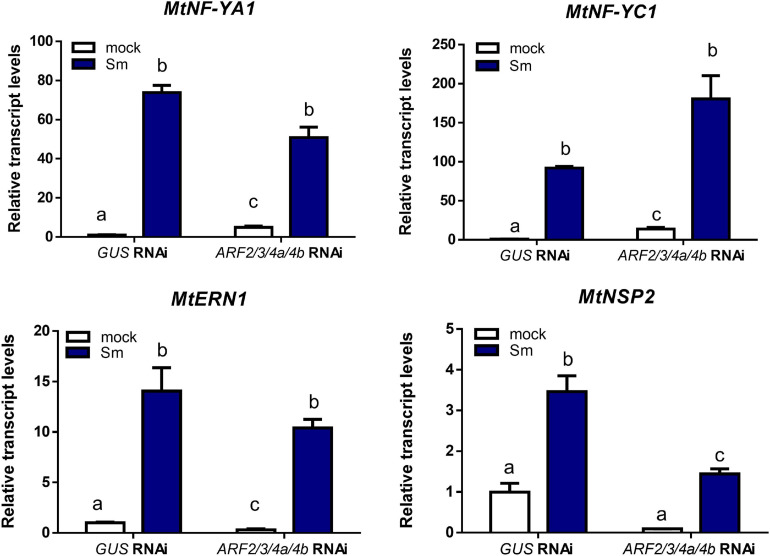
Simultaneous knockdown of *MtARF2, MtARF3, MtARF4a*, and *MtARF4b* interfered with up-regulation of *NSP2* by rhizobia inoculation. Transcript levels of early nodulation markers *MtNF-YA1*, *MtNF-YC1*, *MtERN1*, and *MtNSP2* in *GUS* and *ARF2/3/4a/4b* RNAi roots at 48 h post-inoculation (hpi) with *S. meliloti* (Sm, blue bars) or water (mock, white bars) were determined by RT-qPCR, normalized to *MtHIS3L*, and expressed relative to the *GUS* RNAi mock sample. Different letters indicate statistically significant differences in an unpaired two-tailed Student’s *t* test with a *p* value ≤ 0.05.

Since silencing of *MtARF2, MtARF3, MtARF4a*, and *MtARF4b* or mutations in *MtARF4a* affected nodule formation and bacterial infection, we tested whether the expression of key genes of the nodulation signaling pathway is affected in plants with reduced levels of *MtARF2*, *MtARF3*, and *MtARF4a/b* transcripts (Figure 6). Transcript levels of the A and C subunits of the heterotrimeric transcription factor NF-Y accumulated to significantly higher levels in response to *S. meliloti* (>60- and >100-fold induction for *MtNF-YA1* and *MtNF-YC1*, respectively) with no significant differences between *ARF2/3/4a/4b* RNAi and *GUS* RNAi roots. A similar scenario was found for transcripts of *MtERN1*, which accumulated more than 10-fold upon inoculation with *S. meliloti* as compared to mock in both *ARF2/3/4a/4b* RNAi and *GUS* RNAi roots. However, mRNA levels of the GRAS transcription factor nodulation signaling pathway 2 (MtNSP2) accumulated to a significantly lower extent in *ARF2/3/4a/4b* RNAi roots than in *GUS* RNAi roots after inoculation with *S. meliloti*, indicating that MtARF2, MtARF3, MtARF4a, and/or MtARF4b transcription factors might be required for full activation of *MtNSP*2, in agreement with that previously reported by Hobecker et al. (2017).

### Knockdown of *MtARF2, MtARF3, MtARF4a*, and *MtARF4b* and Mutations in *MtARF4a* Alter Root Architecture

**FIGURE 7:**
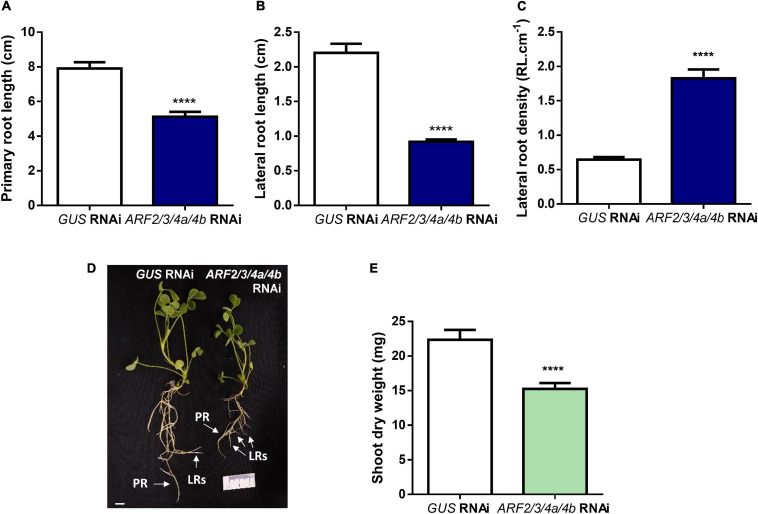
Knockdown of *MtARF2, MtARF3, MtARF4a*, and *MtARF4b* altered root architecture. **(A–C)** Length of independent primary transgenic roots **(A)** and lateral root **(B)** and density of lateral roots **(C)** were measured in *GUS* and *ARF2/3/4a/b* RNAi roots at 15 days after transplantation to square petri dishes slanted agar-Fahraeus medium supplemented with 8 mM KNO_3_. Error bars represent mean ± SE of three independent biological replicates with more than 20 roots in each experiment. Four asterisks indicate statistically significant differences between *GUS* RNAi and *ARF2/3/4a/4b* RNAi roots in an unpaired two-tailed Student’s *t* test with a *p* value ≤ 0.0001. **(D)** Image of *GUS* and *ARF2/3/4a/4b* RNAi composite plants illustrating the shorter primary and lateral roots observed in *ARF2/3/4a/4b* RNAi roots. Each root that emerged directly from the sectioned site of the non-transgenic roots inoculated with *A. rhizogenes* was considered independent transgenic primary root (PR), whereas first-order roots that emerged from each independent transgenic primary root were considered lateral roots (LRs) in the hairy root system. Scale bar: 1 cm. **(E)** Shoot dry weight measured in *GUS* and *ARF2/3/4a/4b* RNAi composite plants at 15 days after transplantation to square petri dishes slanted agar-Fahraeus medium supplemented with 8 mM KNO_3_. Error bars represent mean ± SE of three independent biological replicates with more than 10 composite plants in each experiment. Four asterisks indicate statistically significant differences between *GUS* and *ARF2/3/4a/4b* RNAi composite plants in an unpaired two-tailed Student’s *t* test with a *p* value ≤ 0.0001.

**FIGURE 8:**
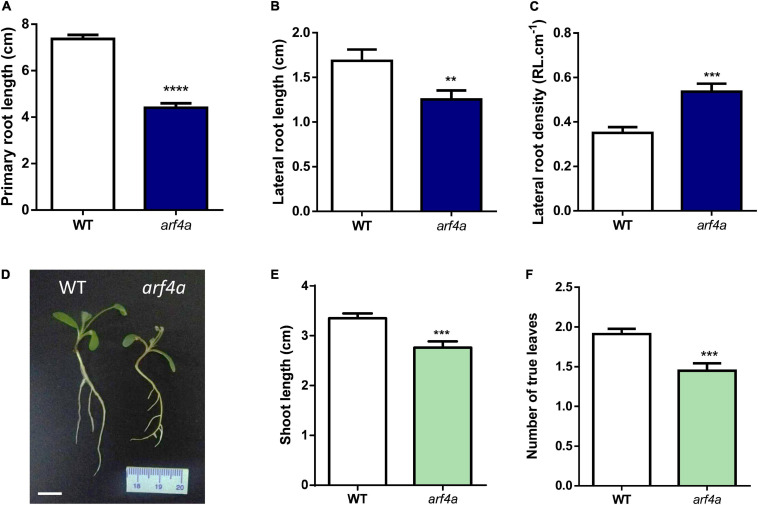
The *arf4a* mutant exhibited altered root and shoot development. **(A–C)** Primary root length **(A)**, lateral root length **(B)**, and lateral root density **(C)** were measured in WT and *arf4a* mutant plants at 7 days after germination (dag). Plants were grown on slanted agar-Fahraeus medium supplemented with 8 mM KNO_3._ Error bars represent mean ± SE of three biological replicates, each with at least 10 plants. Asterisks denote statistically significant differences between WT and *arf4a* plants in an unpaired two-tailed Student’s *t* test (^∗∗^*p* ≤ 0.01, ^∗∗∗^*p* ≤ 0.001, and ^****^p ≤ 0.0001). **(D)** Image of WT and *arf4a* mutant plants at 7 dag illustrating the shorter primary and lateral roots observed in *arf4a* mutant roots. Scale bar: 1 cm. **(E,F)** Shoot length **(E)** and number of true leaves **(F)** measured in WT and *arf4a* mutant at 7 dag. Plants were grown on slanted agar-Fahraeus medium supplemented with 8 mM KNO_3_. Error bars represent mean ± SE of three biological replicates, each with at least 10 plants. Three asterisks denote a statistically significant difference between WT and *arf4a* plants in an unpaired two-tailed Student’s *t* with a *p* value ≤ 0.001.

Recent studies have evidenced that genes required for lateral root development were co-opted for the nodulation program in legume plants, including those involved in auxin biosynthesis, signaling, and responses (Schiessl et al., 2019; Soyano et al., 2019). Thus, we investigated whether *MtARF2*, *MtARF3*, and *MtARF4a/b* members play a role in the control of root development in *M. truncatula* by analyzing the architecture of roots with reduced levels of *MtARF2*, *MtARF3*, and *MtARF4a/b* transcripts. Root architecture of *ARF2/3/4a/4b* RNAi roots was severely affected when plants were grown for 15 days under nitrogen availability. *ARF2/3/4a/4b* RNAi caused a significant reduction in the length of primary and lateral root but enhanced by more than twofold the lateral root density—i.e., the number of lateral roots per primary root centimeter—as compared with control *GUS* RNAi roots (Figures 7A–D). In addition, the dry weight of the shoot was reduced in these plants, most likely because of the limited growth of primary and lateral roots (Figure 7E). In accordance, *arf4a* mutants also exhibited a pronounced and significant reduction in primary and lateral root length as well as a significant increase in lateral root density as compared with WT plants at 7 and 15 dag when grown in the presence of nitrogen (Figures 8A–C and Supplementary Figures 6A–C, respectively). This indicates that *MtARF4a* might act as a modulator that promotes the elongation of primary and lateral roots but limits the formation of new lateral root in *M. truncatula*. On the other hand, *arf4a* mutant plants exhibited a pleiotropic phenotype in the aerial part (Figure 8D), with a significant reduction in shoot length (Figure 8E and Supplementary Figure 6D) and the number of true leaves (Figure 8F and Supplementary Figure 6E). Moreover, in plants of 15 dag, the shape and organ separation of the trifoliate leaves was also affected in *arf4a* mutants, with leaflets closer to each other as compared with WT plants (Supplementary Figure 6F). These results indicate that mutation in *MtARF4a* not only altered the root architecture, but also the shoot development in *M. truncatula*. Altered compound leaf patterning was previously observed in *M. truncatula ago7* mutants, which exhibited reduced levels of *MtARF2, MtARF3*, and *MtARF4a/b* (Zhou et al., 2013), as well as in plants that overexpresses *MtARF3* (Peng et al., 2017). Based on the results presented here, *MtARF4a* might have a direct role in shoot development, or alternatively, the shoot developmental defects observed in *arf4a* mutant plants might be the consequence of the altered growth of primary and lateral roots.

## Discussion

### ARF-Mediated Control of Nodule Organogenesis and Root Architecture

Auxin biosynthesis, signaling, and response are crucial for the developmental programs that control root architecture and the formation of symbiotic nodules (Overvoorde et al., 2010; Lin et al., 2020). Different lines of evidence revealed that there are extensive overlaps in the signaling components and developmental processes that lead to the formation of both types of organs, including those activated by auxin; however, there are also remarkable differences (reviewed by Kohlen et al., 2018). Different ARF members modulate root architecture in legume and non-legume species, some of which promote root development, while others exert inhibitory effects in root development. Here, we found that *M. truncatula* roots with reduced levels of *MtARF2, MtARF3, MtARF4a*, and *MtARF4b* or an *arf4a* single mutant exhibited shorter primary and lateral roots but enhanced lateral root density (Figures 7, 8), suggesting that these ARFs might function in promoting primary and lateral root growth and inhibiting the inception of lateral roots. We have previously shown that reduction of *MtARF2, MtARF3, MtARF4a*, and *MtARF4b* levels produced by overexpression of miR390 promotes elongation of lateral roots without affecting primary root length or lateral root density (Hobecker et al., 2017). This suggests either that miR390 mediates lateral root growth by acting through a pathway that is independent of *MtARF2, MtARF3, MtARF4a*, and *MtARF4b* or that the drastic reduction in the three *ARF* transcript levels caused by RNAi (70–90% depending on the *ARF* mRNA) or null levels of *MtARF4a* results in a distinct and more severe root phenotype than activation of the miR390/*TAS3* pathway by overexpression of miR390, which reduced *ARF* mRNA levels by only 50–80%. The phenotype observed here in *M. truncatula* contrasts with that previously described by Marin et al. (2010) in Arabidopsis, where the authors showed that plants expressing an artificial miRNA that simultaneously knock down the three ARFs (aMIR-ARFs) exhibited longer lateral roots. In addition, individual *arf2*, *arf3*, and *arf4* Arabidopsis mutants showed a mild enhancement in primary and lateral root length as compared to WT plants, whereas *arf3* and *arf4* mutants had reduced lateral root density (Marin et al., 2010). On the other hand, expression of an RNAi that targets *PtARF4* in *P. trichocarpa* increased both lateral root length and density under normal or salt stress conditions (He et al., 2018), whereas overexpression of *OsTAS3* in *O. sativa*, which results in a reduction in ARF levels, yielded denser lateral roots (Lu et al., 2018). Thus, it seems that distinct plant species respond with different alteration of root architecture to the reduction or loss of *ARF2, ARF3*, and/or *ARF4*. These species-specific determination mechanisms have been previously observed in leaf development, where alteration in the production of tasiARFs results in highly variable phenotypic response depending on the species such as wiry leaves in tomato (Yifhar et al., 2012), cylindrical leaves in *O. sativa* (Douglas et al., 2010), reduced number of leaflets in *L. japonicus* (Yan et al., 2010), and lobed leaf margin and widely spaced lateral shoot organs in *M. truncatula* (Zhou et al., 2013). Species-specific phenotypic variation in root architecture has also been observed in response to the lack or reduction of *ARF10/16/17* levels. In Arabidopsis, an *arf10/arf16* double mutant or a miR160-overexpressing line exhibited more lateral roots and reduced primary root growth (Mallory et al., 2005; Wang et al., 2005), whereas reduction of *MtARF10/16/17* levels by overexpression of miR160 in *M. truncatula* did not alter lateral root formation or elongation but instead affected primary root growth by altering the organization of the root apical meristem (Bustos-Sanmamed et al., 2013).

In the root nodule symbiosis, members of the ARF family exert either positive or negative roles on nodule number and rhizobial infection. *GmARF8a* and *GmARF8b* function as negative regulators of nodule formation in *G. max* (Wang et al., 2015). On the other hand, *GmARF10*, *GmARF16*, and *GmARF17*, which are post-transcriptionally repressed by miR160, acts as positive regulators of nodule formation in both determinate and indeterminate nodule-forming species *G. max* (Turner et al., 2013; Nizampatnam et al., 2015) and *M. truncatula* (Bustos-Sanmamed et al., 2013), respectively. Here, we showed that the *MtARF2, MtARF3, MtARF4a*, and *MtARF4b* members of the B clade of the ARF family are required for proper nodule formation and development in *M. truncatula* (Table 1 and Figures 4B,C). This indicates that in addition to members of the C clade, such as *MtARF10, MtARF16*, and *MtARF17*, *MtARF2, MtARF3*, and *MtARF4* can act as positive regulators of indeterminate nodule formation. These results are in agreement with those previously obtained by overexpression of miR390 in *M. truncatula* (Hobecker et al., 2017). Although our RNAi strategy effectively silenced *MtARF2, MtARF3*, and *MtARF4a/b*, expression data indicated that *MtARF4a* was the main ARF transcriptionally activated by rhizobia among these three ARF members (Figure 2 and Supplementary Figure 3). Unfortunately, the high similarity of nucleotide sequences of *MtARF2*, *MtARF3, MtARF4a*, and *MtARF4b* transcripts made it unfeasible to design RNAi constructs specific for each member, preventing us to dissect the individual contribution of *MtARF2*, *MtARF3*, and *MtARF4b* to the root architecture and nodulation phenotype. However, the single *arf4a* mutant showed a very similar nodulation phenotype than that observed by simultaneous silencing of *ARF2/3/4a/4b* (Figures 4B, 5B), suggesting that *MtARF4a* has a preponderant function in nodule formation. This reduction in nodule number observed in *ARF2/3/4a/4b* RNAi roots or in the *arf4a* mutant might be due to a decrease in auxin signaling and response, which is also correlated with the reactivation of cell divisions in the root cortex that promote nodule organogenesis (Kohlen et al., 2018). Intriguingly, it has been suggested that *LjARF2, LjARF3*, and *LjARF4* can potentially act as negative regulators of nodulation in *L. japonicus*, since their expression is enhanced in *reduced leaflet3/argonaute7* (*rel3*/*ago7*) mutants, which are impaired in nodule formation (Li et al., 2014). If this is the case, the contrasting results found in *L. japonicus* (Li et al., 2014) and *M. truncatula* (Hobecker et al., 2017 and this study) suggest that the role of these ARFs in nodulation might be also subjected to species-specific determinant mechanisms. Further analysis in other legume species will certainly help to elucidate whether the mode of action of these ARFs is different in determinate and indeterminate nodule-forming species.

As for other *ARF* genes involved in both lateral root architecture and nodule formation (e.g., *ARF8* and *ARF10/16/17*), the possibility that the symbiotic phenotype observed in *arf4a* mutants might be a consequence of alteration in root architecture and/or shoot development cannot be excluded. However, the nodulation phenotype of *arf4a* mutants—with over 70% reduction in nodule number as compared to WT (Figure 5B)—was more severe than the root architecture phenotype, where lateral root length was reduced by 20% but the number of lateral roots increased by 35% as compared to WT (Figure 8). Considering that nodules formed mainly in lateral roots, the total lateral root system for nodule formation is not drastically altered in *arf4a* mutants; thus, it seems unlikely that root architecture was the cause of the symbiotic phenotype. Remarkably, infection by rhizobia, which can be genetically separated from nodule organogenesis (Oldroyd et al., 2011), was also severely impaired in *arf4a* mutants (Figures 5C,D), supporting a role for *MtARF4a* in the root nodule symbiosis.

### ARFs and Their Role in Rhizobial Infection

Infection by rhizobia can be arrested either at the initiation stage or during the progression of the infection events toward the dividing cortical cells that will form the nodule primordium. Here, we found that silencing of *MtARF2, MtARF3, MtARF4a*, and *MtARF4b* or mutation of *MtARF4a* in *M. truncatula*, resulted in a moderate reduction in the density of infection events, but a severe impairment of the progression of these events to root cortex (Figures 4D,E, 5C,D). The infection phenotype observed here using an RNAi strategy or the *arf4a* mutant was more pronounced than that observed by Hobecker et al. (2017), since overexpression of miR390 reduced the density of infection events but not their progression. This might be explained considering that the RNAi is more effective in reducing *ARF* transcript levels than overexpression of miR390 (70–90 vs. 50–80% depending on the *ARF*). Previously, Breakspear et al. (2014) have shown that mutations in *MtARF16a* also affected infection by rhizobia in *M. truncatula*, mostly caused by a decrease in the formation of pockets containing microcolonies and elongating ITs. Thus, *MtARF16* seems to be required for the initiation of infection rather than for elongation and ramification of ITs. Our results indicate that *MtARF4a* participate not only in the initiation but also in the elongation and ramification of ITs, since the majority of infection events were arrested at the microcolony stage and only a minor proportion of the ITs reached the dividing cortex (Figure 5D). A recent study in *L. japonicus* using a *DR5:GUS* reporter or a LjDII auxin sensor—consisting of the DII domain of Arabidopsis AtIAA28 and nuclear−localized triple YFP—has demonstrated that auxins accumulate specifically in rhizobium-infected root hairs and, moreover, that this accumulation is dependent on the NF signaling (Nadzieja et al., 2018). In addition, the same study revealed that components of the auxin biosynthetic pathway are up-regulated specifically in the rhizobia infected root hairs. In *M. truncatula*, inoculation with *S. meliloti* induced *DR5:GUS* reporter in both infected and uninfected root hairs over the entire infection zone and up-regulated a number of genes involved in auxin signaling and response such as *MtGH3*, *MtSAUR1*, and *MtARF16a* (Breakspear et al., 2014). The promoter*:GFP* analysis presented here has shown that *MtARF4a*, but not *MtARF3*, is expressed in the infected root hairs and in epidermal surrounding cells (Figure 2), supporting the notion that auxin signaling and response have a crucial role in promoting infection events in epidermal cells. This requirement for auxin signaling might be related to changes in cell-cycle progression that increase extensibility of the cell wall during IT formation in the root hairs (Breakspear et al., 2014; Lin et al., 2020).

### ARFs and the Nod Factor Signaling Pathway

We have found that the expression of *MtARF2*, *MtARF3*, and *MtARF4/b* was activated in response to NFs, and that this activation requires the Nod Factor Receptor MtNFP and the MtNF-YA1 transcription factor (Figure 3), suggesting that this set of ARFs might act downstream of NF-Y in the NF signaling pathway. *MtNFP* was also required for timely activation of miR160 in *M. truncatula*, which targets *MtARF10*, *MtARF16*, and *MtARF17* (Bustos-Sanmamed et al., 2013). Conversely, NF perception-dependent expression has been described for the miR167 in *G. max*, which targets *GmARF8a/b*, since accumulation of this miRNA in response to rhizobia was impaired in the non-nodulating mutant defective in the NF receptor *GmNFR1*α (Wang et al., 2015). Thus, it seems that modulation of the expression of different members of the ARFs family is dependent on NF signaling pathway in legumes. In addition, silencing of *MtARF2, MtARF3, MtARF4a*, and *MtARF4b* did not affect the up-regulation of *MtNF-YA1* and *MtNF-YC1* in response to rhizobia (Figure 6), supporting the notion that these ARF members act downstream of the NF-Y complex. On the other hand, we have found that *MtARF2, MtARF3, MtARF4a*, and *MtARF4b* are required for full induction of *MtNSP2* in response to *S. meliloti* (Figure 6), indicating that these ARFs might intercept the Nod signaling pathway acting upstream of MtNSP2. This agrees with previous results that showed that roots overexpressing miR390, which have reduced levels of *MtARF2, MtARF3, MtARF4a*, and *MtARF4b*, failed to up-regulate *MtNSP1* and *MtNSP2* in *M. truncatula* in response to rhizobia (Hobecker et al., 2017). In *G. max*, GmARF8a/b can potentially act as negative regulators of *GmNSP1* and other symbiotically related genes (Wang et al., 2015). On the other hand, *G. max* roots with reduced levels of miR160, which targets *ARF10/16/17*, exhibited greater induction of *GmNSP1* upon inoculation with its symbiotic partner *Bradyrhizobium japonicum*, indicating that *GmARF10*, *GmARF16*, and *GmARF17* can act as positive regulators of *GmNSP1* (Nizampatnam et al., 2015). These results indicate that different ARF members intercept the nodulation signaling pathway acting either as positive or negative regulators of distinct components required for nodulation, such as *NSP1* and *NSP2*. Further analysis will help to elucidate whether ARFs can act as direct activators or repressors of *NSP1* and *NSP2*. Interestingly, recent studies on the Arabidopsis AtARF5 revealed a new paradigm to explain the ARF-mediated transcriptional response to auxin. In addition to the auxin−mediated degradation of Aux/IAA proteins that releases ARF repression, ARFs can potentially act as pioneer transcription factors by recruiting chromatin remodeling proteins that promote a chromatin permissive configuration at auxin-regulated loci (e.g., *PLETHORA*), allowing proximal *cis*−AuxREs to become accessible to form higher-order transcriptional complexes, and adding a new layer of complexity to the auxin transcriptional response (Kornet and Scheres, 2009; Wu et al., 2015; Chandler, 2016). Undoubtedly, more research is needed to clarify which loci are targeted by *MtARF2, MtARF3, MtARF4a*, and *MtARF4b* and the mechanisms by which these ARFs mediate auxin response in the root nodule symbiosis. The results presented here regarding the positive role of these ARF members in nodulation and its crosstalk with the nodulation signaling pathway represent an initial step toward the elucidation of such mechanisms.

## Data Availability Statement

The raw data supporting the conclusions of this article will be made available by the authors, without undue reservation.

## Author Contributions

MZ and FB conceived the research, conceptualized the study, and supervised the study. CK, KH, MZ, FB, and AN designed the experiments. CK, KH, and AN performed the experiments. JW, KM, and AN contributed to the biological material. CK, KH, FB, and MZ analyzed the data. CK, MZ, and FB wrote the original draft of the article. CK, KH, AN, FB, and MZ reviewed and edited the article. AN, FB, and MZ acquired funding. All authors contributed to the article and approved the submitted version.

## Conflict of Interest

JW and KM were employed by the company Noble Research Institute LLC. The remaining authors declare that the research was conducted in the absence of any commercial or financial relationships that could be construed as a potential conflict of interest.

## Footnotes

1 http://www.montpellier.inra.fr

2 https://www.arabidopsis.org/

3 https://medicago.toulouse.inra.fr/MtrunA17r5.0-ANR/

4 https://www.ebi.ac.uk/Tools/msa/clustalo/

5 http://sourceforge.net/projects/boxshade/

## References

[B1] Amor B. B. Shaw S. L. Oldroyd G. E. Maillet F. Penmetsa R. V. Cook D. (2003). The NFP locus of *Medicago truncatula* controls an early step of Nod factor signal transduction upstream of a rapid calcium flux and root hair deformation. *Plant J.* 34 495–506. 10.1046/j.1365-313x.2003.01743.x 12753588

[B2] Ariel F. Diet A. Verdenaud M. Gruber V. Frugier F. Chan R. (2010). Environmental regulation of lateral root emergence in *Medicago truncatula* requires the HD-Zip I transcription factor HB1. *Plant Cell* 22 2171–2183. 10.1105/tpc.110.074823 20675575 PMC2929095

[B3] Baudin M. Laloum T. Lepage A. Ripodas C. Ariel F. Frances L. (2015). A phylogenetically conserved group of nuclear factor-Y transcription factors interact to control nodulation in legumes. *Plant Physiol.* 169 2761–2773. 26432878 10.1104/pp.15.01144 PMC4677902

[B4] Bishopp A. Bennett M. J. (2019). Turning lateral roots into nodules. *Science* 366 953–954. 10.1126/science.aay8620 31753986

[B5] Blanco F. A. Meschini E. P. Zanetti M. E. Aguilar O. M. (2009). A small GTPase of the Rab family is required for root hair formation and preinfection stages of the common bean-Rhizobium symbiotic association. *Plant Cell* 21 2797–2810. 10.1105/tpc.108.063420 19749154 PMC2768941

[B6] Boisson-Dernier A. Chabaud M. Garcia F. Becard G. Rosenberg C. Barker D. G. (2001). Agrobacterium rhizogenes-transformed roots of *Medicago truncatula* for the study of nitrogen-fixing and endomycorrhizal symbiotic associations. *Mol. Plant Microbe Interact.* 14 695–700. 10.1094/mpmi.2001.14.6.695 11386364

[B7] Breakspear A. Liu C. Roy S. Stacey N. Rogers C. Trick M. (2014). The root hair “infectome” of *Medicago truncatula* uncovers changes in cell cycle genes and reveals a requirement for Auxin signaling in rhizobial infection. *Plant Cell* 26 4680–4701. 10.1105/tpc.114.133496 25527707 PMC4311213

[B8] Bustos-Sanmamed P. Mao G. Deng Y. Elouet M. Khan G. A. Bazin J. (2013). Overexpression of miR160 affects root growth and nitrogen-fixing nodule number in *Medicago truncatula*. *Funct. Plant Biol.* 40 1208–1220. 10.1071/fp13123 32481189

[B9] Chandler J. W. (2016). Auxin response factors. *Plant Cell Environ.* 39 1014–1028. 10.1111/pce.12662 26487015

[B10] Cheng X. Wang M. Lee H. K. Tadege M. Ratet P. Udvardi M. (2014). An efficient reverse genetics platform in the model legume *Medicago truncatula*. *New Phytol.* 201 1065–1076. 24206427 10.1111/nph.12575

[B11] Combier J. P. Frugier F. De Billy F. Boualem A. El-Yahyaoui F. Moreau S. (2006). MtHAP2-1 is a key transcriptional regulator of symbiotic nodule development regulated by microRNA169 in *Medicago truncatula*. *Genes Dev.* 20 3084–3088. 10.1101/gad.402806 17114582 PMC1635144

[B12] De Smet I. Lau S. Voss U. Vanneste S. Benjamins R. Rademacher E. H. (2010). Bimodular auxin response controls organogenesis in *Arabidopsis*. *Proc. Natl. Acad. Sci. U.S.A.* 107 2705–2710. 10.1073/pnas.0915001107 20133796 PMC2823897

[B13] Douglas R. N. Wiley D. Sarkar A. Springer N. Timmermans M. C. P. Scanlon M. J. (2010). *Ragged seedling2* encodes an ARGONAUTE7-like protein required for mediolateral expansion, but not dorsiventrality, of maize leaves. *Plant Cell* 22 1441–1451. 10.1105/tpc.109.071613 20453116 PMC2899878

[B14] Doyle J. J. (2011). Phylogenetic perspectives on the origins of nodulation. *Mol. Plant Microbe Interact.* 24 1289–1295. 10.1094/mpmi-05-11-0114 21995796

[B15] Fahraeus G. (1957). The infection of clover root hairs by nodule bacteria studied by a simple glass slide technique. *J. Gen. Microbiol.* 16 374–381. 10.1099/00221287-16-2-374 13416514

[B16] Felsenstein J. (1985). Confidence limits on phylogenies: an approach using the bootstrap. *Evolution* 39 783–791. 10.2307/2408678 28561359

[B17] Finet C. Berne-Dedieu A. Scutt C. P. Marlétaz F. (2013). Evolution of the ARF gene family in land plants: old domains, new tricks. *Mol. Biol. Evol.* 30 45–56. 10.1093/molbev/mss220 22977118

[B18] Franssen H. J. Xiao T. T. Kulikova O. Wan X. Bisseling T. Scheres B. (2015). Root developmental programs shape the *Medicago truncatula* nodule meristem. *Development* 142 2941–2950. 10.1242/dev.120774 26253408

[B19] Guilfoyle T. J. Hagen G. (2007). Auxin response factors. *Curr. Opin. Plant Biol.* 10 453–460. 17900969 10.1016/j.pbi.2007.08.014

[B20] He F. Xu C. Fu X. Shen Y. Guo L. Leng M. (2018). The MicroRNA390/TRANS-acting short interfering RNA3 module mediates lateral root growth under salt stress via the auxin pathway. *Plant Physiol.* 177 775–791. 10.1104/pp.17.01559 29717017 PMC6001319

[B21] Herrbach V. Rembliere C. Gough C. Bensmihen S. (2014). Lateral root formation and patterning in *Medicago truncatula*. *J. Plant Physiol.* 171 301–310. 10.1016/j.jplph.2013.09.006 24148318

[B22] Hobecker K. V. Reynoso M. A. Bustos-Sanmamed P. Wen J. Mysore K. S. Crespi M. (2017). The MicroRNA390/TAS3 pathway mediates symbiotic nodulation and lateral root growth. *Plant Physiol.* 174 2469–2486. 10.1104/pp.17.00464 28663332 PMC5543954

[B23] Karimi M. Depicker A. Hilson P. (2007). Recombinational cloning with plant gateway vectors. *Plant Physiol.* 145 1144–1154. 10.1104/pp.107.106989 18056864 PMC2151728

[B24] Karimi M. Inze D. Depicker A. (2002). GATEWAY vectors for *Agrobacterium*-mediated plant transformation. *Trends Plant Sci.* 7 193–195. 10.1016/s1360-1385(02)02251-3 11992820

[B25] Kohlen W. Ng J. L. P. Deinum E. E. Mathesius U. (2018). Auxin transport, metabolism, and signaling during nodule initiation: indeterminate and determinate nodules. *J. Exp. Bot.* 69 229–244. 10.1093/jxb/erx308 28992078

[B26] Kornet N. Scheres B. (2009). Members of the GCN5 histone acetyltransferase complex regulate PLETHORA-mediated root stem cell niche maintenance and transit amplifying cell proliferation in *Arabidopsis*. *Plant Cell* 21 1070–1079. 10.1105/tpc.108.065300 19376933 PMC2685635

[B27] Kumar S. Stecher G. Li M. Knyaz C. Tamura K. (2018). MEGA X: molecular evolutionary genetics analysis across computing platforms. *Mol. Biol. Evol.* 35 1547–1549. 10.1093/molbev/msy096 29722887 PMC5967553

[B28] Laporte P. Lepage A. Fournier J. Catrice O. Moreau S. Jardinaud M. F. (2014). The CCAAT box-binding transcription factor NF-YA1 controls rhizobial infection. *J. Exp. Bot.* 65 481–494. 10.1093/jxb/ert392 24319255 PMC3904707

[B29] Li X. Lei M. Yan Z. Wang Q. Chen A. Sun J. (2014). The REL3-mediated TAS3 ta-siRNA pathway integrates auxin and ethylene signaling to regulate nodulation in *Lotus japonicus*. *New Phytol.* 201 531–544. 10.1111/nph.12550 24164597

[B30] Lin J. Frank M. Reid D. (2020). No home without hormones: how plant hormones control legume nodule organogenesis. *Plant Commun*. 1:100104. 10.1016/j.xplc.2020.100104 33367261 PMC7747975

[B31] Lu Y. Feng Z. Liu X. Bian L. Xie H. Zhang C. (2018). MiR393 and miR390 synergistically regulate lateral root growth in rice under different conditions. *BMC Plant Biol.* 18:261. 10.1186/s12870-018-1488-x 30373525 PMC6206659

[B32] Mallory A. C. Bartel D. P. Bartel B. (2005). MicroRNA-directed regulation of *Arabidopsis* AUXIN RESPONSE FACTOR17 is essential for proper development and modulates expression of early auxin response genes. *Plant Cell* 17 1360–1375. 10.1105/tpc.105.031716 15829600 PMC1091760

[B33] Marin E. Jouannet V. Herz A. Lokerse A. S. Weijers D. Vaucheret H. (2010). miR390, *Arabidopsis* TAS3 tasiRNAs, and their AUXIN RESPONSE FACTOR targets define an autoregulatory network quantitatively regulating lateral root growth. *Plant Cell* 22 1104–1117. 10.1105/tpc.109.072553 20363771 PMC2879756

[B34] Meade H. M. Signer E. R. (1977). Genetic mapping of *Rhizobium meliloti*. *Proc. Natl. Acad. Sci. U.S.A.* 74 2076–2078. 10.1073/pnas.74.5.2076 266730 PMC431077

[B35] Middleton P. H. Jakab J. Penmetsa R. V. Starker C. G. Doll J. Kalo P. (2007). An ERF transcription factor in *Medicago truncatula* that is essential for Nod factor signal transduction. *Plant Cell* 19 1221–1234. 10.1105/tpc.106.048264 17449807 PMC1913751

[B36] Nadzieja M. Kelly S. Stougaard J. Reid D. (2018). Epidermal auxin biosynthesis facilitates rhizobial infection in *Lotus japonicus*. *Plant J.* 95 101–111. 10.1111/tpj.13934 29676826

[B37] Nei M. Kumar S. (2000). *Molecular Evolution and Phylogenetics.* New York, NY: Oxford University Press.

[B38] Nizampatnam N. R. Schreier S. J. Damodaran S. Adhikari S. Subramanian S. (2015). microRNA160 dictates stage-specific auxin and cytokinin sensitivities and directs soybean nodule development. *Plant J.* 84 140–153. 10.1111/tpj.12965 26287653

[B39] Okushima Y. Overvoorde P. J. Arima K. Alonso J. M. Chan A. Chang C. (2005). Functional genomic analysis of the AUXIN RESPONSE FACTOR gene family members in *Arabidopsis thaliana*: unique and overlapping functions of ARF7 and ARF19. *Plant Cell* 17 444–463. 10.1105/tpc.104.028316 15659631 PMC548818

[B40] Oldroyd G. E. Murray J. D. Poole P. S. Downie J. A. (2011). The rules of engagement in the legume-rhizobial symbiosis. *Annu. Rev. Genet.* 45 119–144. 10.1146/annurev-genet-110410-132549 21838550

[B41] Osipova M. A. Mortier V. Demchenko K. N. Tsyganov V. E. Tikhonovich I. A. Lutova L. A. (2012). Wuschel-related homeobox5 gene expression and interaction of CLE peptides with components of the systemic control add two pieces to the puzzle of autoregulation of nodulation. *Plant Physiol.* 158 1329–1341. 10.1104/pp.111.188078 22232385 PMC3291250

[B42] Overvoorde P. Fukaki H. Beeckman T. (2010). Auxin control of root development. *Cold Spring Harb. Perspect. Biol.* 2:a001537. 10.1101/cshperspect.a001537 20516130 PMC2869515

[B43] Pecrix Y. Staton S. E. Sallet E. Lelandais-Briere C. Moreau S. Carrere S. (2018). Whole-genome landscape of *Medicago truncatula* symbiotic genes. *Nat. Plants* 4 1017–1025. 30397259 10.1038/s41477-018-0286-7

[B44] Peng J. Berbel A. Madueño F. Chen R. (2017). AUXIN response FACTOR3 regulates compound leaf patterning by directly repressing PALMATE-LIKE PENTAFOLIATA1 expression in *Medicago truncatula*. *Front. Plant Sci.* 8:1630. 10.3389/fpls.2017.01630 28979286 PMC5611443

[B45] Penmetsa R. V. Cook D. R. (1997). A legume ethylene-insensitive mutant hyperinfected by its rhizobial symbiont. *Science* 275 527–530. 10.1126/science.275.5299.527 8999796

[B46] Prayitno J. Rolfe B. G. Mathesius U. (2006). The Ethylene-insensitive sickle mutant of *Medicago truncatula* shows altered auxin transport regulation during nodulation. *Plant Physiol.* 142 168–180. 10.1104/pp.106.080093 16844840 PMC1557604

[B47] Quandt H. J. Pühler A. Broer I. (1993). Transgenic root nodules of Vicia hirsuta: a fast and efficient system for the study of gene expression in indeterminate-type nodules. *Mol. Plant Microbe Interact.* 6 699–706. 10.1094/mpmi-6-699

[B48] Reynoso M. A. Blanco F. A. Bailey-Serres J. Crespi M. Zanetti M. E. (2013). Selective recruitment of mRNAs and miRNAs to polyribosomes in response to rhizobia infection in *Medicago truncatula*. *Plant J.* 73 289–301. 10.1111/tpj.12033 23050939

[B49] Saitou N. Nei M. (1987). The neighbor-joining method: a new method for reconstructing phylogenetic trees. *Mol. Biol. Evol.* 4 406–425. 3447015 10.1093/oxfordjournals.molbev.a040454

[B50] Salehin M. Bagchi R. Estelle M. (2015). SCFTIR1/AFB-based auxin perception: mechanism and role in plant growth and development. *Plant Cell* 27 9–19. 10.1105/tpc.114.133744 25604443 PMC4330579

[B51] Schiessl K. Lilley J. L. S. Lee T. Tamvakis I. Kohlen W. Bailey P. C. (2019). Nodule inception recruits the lateral root developmental program for symbiotic nodule organogenesis in *Medicago truncatula*. *Curr. Biol.* 29 3657.e5–3668.e5. 31543454 10.1016/j.cub.2019.09.005 PMC6839406

[B52] Shen C. Yue R. Sun T. Zhang L. Xu L. Tie S. (2015). Genome-wide identification and expression analysis of auxin response factor gene family in *Medicago truncatula*. *Front. Plant Sci.* 6:73. 10.3389/fpls.2015.00073 25759704 PMC4338661

[B53] Shrestha A. Zhong S. Therrien J. Huebert T. Sato S. Mun T. (2020). *Lotus japonicus* nuclear factor YA1, a nodule emergence stage-specific regulator of auxin signalling. *New Phytol.* 229 1535–1552. 10.1111/nph.16950 32978812 PMC7984406

[B54] Smit P. Limpens E. Geurts R. Fedorova E. Dolgikh E. Gough C. (2007). Medicago LYK3, an entry receptor in rhizobial nodulation factor signaling. *Plant Physiol.* 145 183–191. 10.1104/pp.107.100495 17586690 PMC1976573

[B55] Soyano T. Liu M. Kawaguchi M. Hayashi M. (2021). Leguminous nodule symbiosis involves recruitment of factors contributing to lateral root development. *Curr. Opin. Plant Biol.* 59:102000. 10.1016/j.pbi.2020.102000 33454544

[B56] Soyano T. Shimoda Y. Kawaguchi M. Hayashi M. (2019). A shared gene drives lateral root development and root nodule symbiosis pathways in *Lotus*. *Science* 366 1021–1023. 10.1126/science.aax2153 31754003

[B57] Tadege M. Wen J. He J. Tu H. Kwak Y. Eschstruth A. (2008). Large-scale insertional mutagenesis using the Tnt1 retrotransposon in the model legume *Medicago truncatula*. *Plant J.* 54 335–347. 10.1111/j.1365-313x.2008.03418.x 18208518

[B58] Tian C. F. Garnerone A. M. Mathieu-Demaziere C. Masson-Boivin C. Batut J. (2012). Plant-activated bacterial receptor adenylate cyclases modulate epidermal infection in the *Sinorhizobium meliloti*-Medicago symbiosis. *Proc. Natl. Acad. Sci. U.S.A.* 109 6751–6756. 10.1073/pnas.1120260109 22493242 PMC3340038

[B59] Tiwari S. B. Hagen G. Guilfoyle T. (2003). The roles of auxin response factor domains in auxin-responsive transcription. *Plant Cell* 15 533–543. 10.1105/tpc.008417 12566590 PMC141219

[B60] Traubenik S. Reynoso M. A. Hobecker K. Lancia M. Hummel M. Rosen B. (2020). Reprogramming of root cells during nitrogen-fixing symbiosis involves dynamic polysome association of coding and noncoding RNAs. *Plant Cell* 32 352–373. 10.1105/tpc.19.00647 31748328 PMC7008484

[B61] Turner M. Nizampatnam N. R. Baron M. Coppin S. Damodaran S. Adhikari S. (2013). Ectopic expression of miR160 results in auxin hypersensitivity, cytokinin hyposensitivity, and inhibition of symbiotic nodule development in soybean. *Plant Physiol.* 162 2042–2055. 10.1104/pp.113.220699 23796794 PMC3729781

[B62] Ulmasov T. Hagen G. Guilfoyle T. J. (1999). Activation and repression of transcription by auxin-response factors. *Proc. Natl. Acad. Sci. U.S.A.* 96 5844–5849. 10.1073/pnas.96.10.5844 10318972 PMC21948

[B63] Vandesompele J. De Preter K. Pattyn F. Poppe B. Van Roy N. De Paepe A. (2002). Accurate normalization of real-time quantitative RT-PCR data by geometric averaging of multiple internal control genes. *Genome Biol.* 3:research0034.1. 10.1186/gb-2002-3-7-research0034 PMC126239 12184808

[B64] Wang S. Tiwari S. B. Hagen G. Guilfoyle T. J. (2005). AUXIN RESPONSE FACTOR7 restores the expression of auxin-responsive genes in mutant *Arabidopsis* leaf mesophyll protoplasts. *Plant Cell* 17 1979–1993. 10.1105/tpc.105.031096 15923351 PMC1167546

[B65] Wang Y. Li K. Chen L. Zou Y. Liu H. Tian Y. (2015). MicroRNA167-directed regulation of the auxin response factors GmARF8a and GmARF8b is required for soybean nodulation and lateral root development. *Plant Physiol.* 168 984–999. 10.1104/pp.15.00265 25941314 PMC4741323

[B66] Wilmoth J. C. Wang S. Tiwari S. B. Joshi A. D. Hagen G. Guilfoyle T. J. (2005). NPH4/ARF7 and ARF19 promote leaf expansion and auxin-induced lateral root formation. *Plant J.* 43 118–130. 10.1111/j.1365-313x.2005.02432.x 15960621

[B67] Wu M. F. Yamaguchi N. Xiao J. Bargmann B. Estelle M. Sang Y. (2015). Auxin-regulated chromatin switch directs acquisition of flower primordium founder fate. *eLife* 4:e09269. 10.7554/eLife.09269 PMC4600763 26460543

[B68] Xia R. Xu J. Meyers B. C. (2017). The emergence, evolution, and diversification of the miR390-TAS3-ARF pathway in land plants. *Plant Cell* 29 1232–1247. 10.1105/tpc.17.00185 28442597 PMC5502456

[B69] Xiao T. T. Schilderink S. Moling S. Deinum E. E. Kondorosi E. Franssen H. (2014). Fate map of *Medicago truncatula* root nodules. *Development* 141 3517–3528. 10.1242/dev.110775 25183870

[B70] Yan J. Cai X. Luo J. Sato S. Jiang Q. Yang J. (2010). The REDUCED LEAFLET genes encode key components of the trans-acting small interfering RNA pathway and regulate compound leaf and flower development in Lotus japonicus. *Plant Physiol.* 152 797–807. 10.1104/pp.109.140947 19955265 PMC2815879

[B71] Yifhar T. Pekker I. Peled D. Friedlander G. Pistunov A. Sabban M. (2012). Failure of the tomato trans-acting short interfering RNA program to regulate AUXIN RESPONSE FACTOR3 and ARF4 underlies the wiry leaf syndrome. *Plant Cell* 24 3575–3589. 10.1105/tpc.112.100222 23001036 PMC3480288

[B72] Yoon E. K. Yang J. H. Lim J. Kim S. H. Kim S. K. Lee W. S. (2010). Auxin regulation of the microRNA390-dependent transacting small interfering RNA pathway in *Arabidopsis* lateral root development. *Nucleic Acids Res.* 38 1382–1391. 10.1093/nar/gkp1128 19969544 PMC2831332

[B73] Zhou C. Han L. Fu C. Wen J. Cheng X. Nakashima J. (2013). The trans-acting short interfering RNA3 pathway and no apical meristem antagonistically regulate leaf margin development and lateral organ separation, as revealed by analysis of an argonaute7/lobed leaflet1 mutant in *Medicago truncatula*. *Plant Cell* 25 4845–4862. 10.1105/tpc.113.117788 24368797 PMC3903991

