# Supplementary material for: Auxin Response Factor 2 (ARF2), ARF3, and ARF4 Mediate Both Lateral Root and Nitrogen Fixing Nodule Development in Medicago truncatula
Source: Front Plant Sci. 2021 Apr 8;12:659061. doi: 10.3389/fpls.2021.659061 (PMC8060633; doi:10.3389/fpls.2021.659061)
Supplement: Supplementary file 1 [file Data_Sheet_1.PDF]

Supplementary Figure 1

```
MtARF4a     1 MEIDLNNEVIEVEKNALCHKECEKGFCFCV--SCLSPSTCSSSSSTSPLVSSSYLELWHA
MtARF4b     1 MEIDLNDSITEVKKNVCSNGKCEKSVCCVCTLSSSSSPTCSSSSSTSAIVSSSYLELWHA

MtARF4a    59 CAGPLTSLPKKGNVVVYFPQGHLEQFASFSPFKQLEIPNYDLQPQIFCRVVNVQLLANKE
MtARF4b    61 CAGPLTSLPKKGNVVVYFPQGHLEQVASLSLFSSLEIPTYGLQPQILCRVVNVQLLANKE

MtARF4a   119 NDEVYTQVTLLPQAELAGMHMEGKEVEELEGDEEGDGGSPTKSTPHMFCKTLTVSDTSTH
MtARF4b   121 NDEVYTQVALLPQAELAGMCLDDKEPEGLEADDEGNGRSPTKLASHMFCKTLTASDTSTH

MtARF4a   179 GGFSVPRRAAEDCFPPL-------------DYKLQRPSQELVAKDLHGVEWKFRHIYRGQ
MtARF4b   181 GGFSVPRRAAEDCFPPLVQHYEPVWICLFEDYKQQRPSQELVAKDLHGVGWKFRHIYRGQ

MtARF4a   226 PRRHLLTTGWSIFVNQKNLVSGDAVLFLRGQNGELRLGIRRAVRPRNGLPESIVGNQNCY
MtARF4b   241 PRRHLLTTGWSIFVSQKNLVSGDAVLFLRGENGELRLGIRRAARPRNGLPESIIGNQSCS

MtARF4a   286 PNFLSSVANAISTKSMFHVFYSPRASHAEFVVPYQKYVKSIKNPMTIGTRFKMRIEMDES
MtARF4b   301 PSFLSSVANAISAKSMFHVFYSPRASHADFVVPYQKYAKSIRNPVTIGTRFKMKFEMDES

MtARF4a   346 PERRCSSGMLIGINDLDPYRWPKSKWRCLMVRWDDDTETNHQDRVSPWEIDPSSPQPPLS
MtARF4b   361 PERRCSSGIVTGMSDLDPYKWPKSKWRCLMVRWDEDIGANHQDRVSPWEIDPSTSLPPLN

MtARF4a   406 IQSSPRLKKPRTGLLVASPNHLITGMNPNGISGMMGFEESVRSPKVLQGQENTGFMSLYY
MtARF4b   421 IQSSRRLKKLRTGLHVESPSHFITA----GDSGFMDFDESIRSSKVLQGQEKTSFMSLYY

MtARF4a   466 GCDKVTNQPGFELSTSSHHQNLASTGIGKVVTSSELMSVHPFSYAGFMESNNFPRVLQGQ
MtARF4b   477 GCDTVTKQKEFDINSLR-HTNLASNGAR-KITSSEFTRIQPSSYADFTEMNRFPRVLQAQ

MtARF4a   526 EICKLKSLSGKVDFNIGAWGTKP----------------------SFQSACFPYGDIDKS
MtARF4b   535 EIYPLRSLTGKVDLNLNSWGKTNVSYTKYNLHNATKLNFHSLGSEVLQNSSFPYGDIHKV

MtARF4a   564 NQA-SMFSSKHTSFMSDNVPFNTPSIVAGDIRKEVGRSGSNLLPNEHKLQDNVSASASLV
MtARF4b   595 GQGSSMLCSKPTNFQLGNVSFNTPSSQIGALRNEVGLSSFK-IRNEQKLQNDISAATS-L

MtARF4a   623 DTNRNAPNDNNVKGKANSCKLFGFPLSG--EPSSQNLQNTAKRSCTKVHKQGSLVGRAID
MtARF4b   653 DANIRISNDENFKEMVNPCKLFGFSLSAAAETTSQNLQNSAKRSCTKVHKQGSLVGRAID

MtARF4a   681 LSRLSGYNDLLSELEKLFGMEGLLRDSDKGWRILYTDSENDIMVVGDDPWHEFCDVVSKI
MtARF4b   713 LSRLSSYNDLVSELERLFGMEGLLRDPDKGWRILYTDSENDIMVVGDDPWHEFCDMVSKI

MtARF4a   741 HIYTKEEVEKMTFGMMTNDDTHSCLDQAPVIIEPSKSSSVGQPDYSPTVVRV
MtARF4b   773 HIYTQEEVEKMTIGMMN-DDNQSCLEQTPLIMEASKSSSVGQPDSSTTVVRI
```

Supplementary Figure 1. Alignment of MtARF4a and MtARF4b amino acid sequences. The multiple alignment was generated using the Clustal Omega algorithm available at EMBL-EBI (https://www.ebi.ac.uk/Tools/msa/clustalo/) and decorated with BOXSHADE (http://sourceforge.net/projects/boxshade/). Identical amino acids were shaded in black, whereas synonymous substitutions were shaded in gray. Locus IDs are MtrunA17_Chr4g0029671 (Medtr4g060460) and MtrunA17_Chr2g0326281 (Medtr2g093740) for ARF4a and ARF4b, respectively.

Supplementary Figure 2

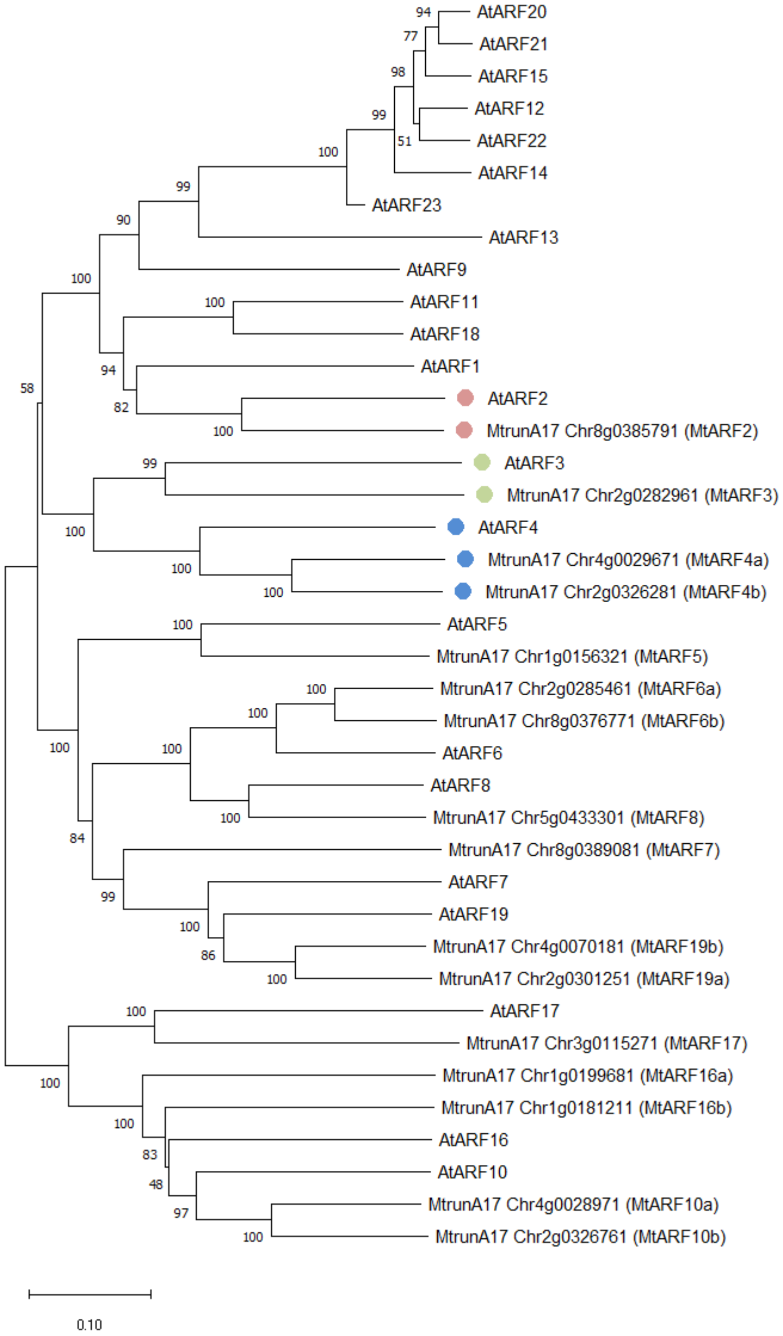

Supplementary Figure 2. Phylogenetic tree of amino acid sequences of Arabidopsis and *M. truncatula* members of the ARF family. All members of the Arabidopsis ARF family were retrieved from the TAIR database (https://www.arabidopsis.org/). *M. truncatula* ARF members linked to root development or nodulation were retrieved from were retrieved from the recently released version of the *M. truncatula* genome MtrunA17r5.0-ANR (https://medicago.toulouse.inra.fr/MtrunA17r5.0-ANR/, Pecrix et al. 2018). The tree was generated using MEGA X (Kumar et al, 2018) using the Neighbor-Joining method. The percentage of replicate trees in which the associated taxa clustered together in the bootstrap test (10000 replicates) is shown next to the branches (Felsentein et al, 1985). The evolutionary distances were computed using the p-distance method (Nei and Kumar, 2000). The ARF2 ARF3, and ARF4 clades were labelled with pink, green and blue circles, respectively.

Supplementary Figure 3

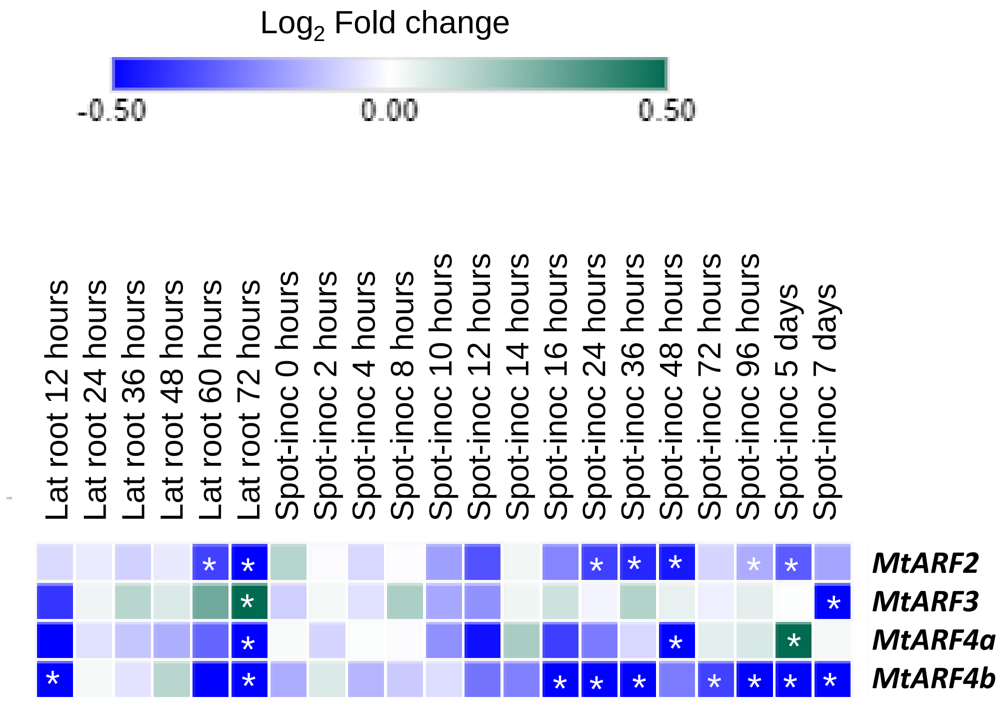

Supplementary Figure 3. Heat map of *MtARF2*, *MtARF3*, *MtARF4a* and *MtARF4b* expression during lateral root and nodule formation. Log_2 Fold change of expression values at indicated time points upon induction of lateral root formation (Lat root) as compared with not induction of lateral roots or upon spot inoculation with droplets of *S. meliloti* suspension (Spot-inoc) as compared with non inoculated roots are presented. Data were retrieved from Schiessl et al. (2019), where genes with Fold changes >1.5 were considered as differentially expressed. The asterisk indicates significant differences at each time point between induced lateral roots not induced roots or between spot inoculated roots and not inoculated roots with an adjusted p value ≤ 0.5.

Supplementary Figure 4

```
MtARF2      1237 AACGTGGTGCCGTCATCCCCAGATTCTTCTGTTCTTACCCGCGAAGCATCGTC----AAA
MtARF3      1225 GGATTCAC------TTCAGCAAAGCTA-GAATTTCCAGT---------TCCCAATGGGAT
MtARF4a     1252 GGTCTACTGGTTGCCTCACCCAATCAC-CTCATCACTGGTATGAATCCTAATGGAATCAG
MtARF4b     1297 GGTCTGCATGTTGAATCACCGAGTCAC-TTTATCACTGC------------TGGAGACAG

                                                      tasiARFs target site
MtARF2      1293 AGTAAGCATGGACCCTTTGCCCACTAGTGGTTTCCAGAGGGTCTTGCAAGGTCAAGAATC
MtARF3      1269 TGGAGCGTCAGACTTTGGGGAATCATTAAGATTCCGAAAGGTCTTGCAAGGTCAAGAAAT
MtARF4a     1311 TGGAATGATGGGTTTTGAGGAGTCAGTAAGATCACCAAAGGTCTTGCAAGGTCAAGAAAA
MtARF4b     1344 TGGGTTTATGGACTTTGATGAGTCTATAAGATCATCCAAGGTCTTGCAAGGTCAAGAAAA

MtARF2      1353 ATCGACCTTGAGAGGAAATTTGGCTGAAAGCAACGACTCTTACACTGCAGAGAAGTCTGT
MtARF3      1329 TATGGGCATGAATACTCCCTATGACAGTATTAATGCT---CAGAGTCCCCGGTT---ATA
MtARF4a     1371 TACAGGTTTTATGTCACTATACTATGGATGTGACAAA---GTAACCAACCAGCCAGGTTT
MtARF4b     1404 AACAAGTTTTATGTCACTATATTATGGATGTGACACA---GTAACTAAGCAGAAAGAATT

MtARF2      1413 TGCGTGGACTCCTGCAACTGATGAAGAAAAGATGGATGCTGTTTCTACTTCAAGAAGGTA
MtARF3      1383 TGAGCTAGGGAGGTGCTATCCTGGTTCAAACTGTTCCGGGATTGCTGCAACAGGAAA---
MtARF4a     1428 TGAGTTGAGCACTTCAAGTCATCATCAAAACCTTGCATCAACTGGAATAGGAAAAGT---
MtARF4b     1461 TGATATCAATTCTCTAAGACATACTA---ATCTTGCATCAAAT---GGAGCAAGAAA---

MtARF2      1473 TGGTTCAGAGAACTGGATGCCAATGTCAAGGCAGGAACCAACGTATTCGGACCTTCTGTC
MtRF3       1440 ---------------TATC---------------------AGAATGCACCCAGCGGCTTC
MtARF4a     1485 ---------------AGTT---------------------ACCTCTTCTGAGCTTATGAG
MtARF4b     1512 ---------------AATT---------------------ACTTCTTCTGAGTTTACGAG

                                                            tasiARFs target site
MtARF2      1533 GGGATTTGGTAGTACCCGTGAAGGCAA-----ACATAACATGCTTACTCAATGGCCTGTA
MtARF3      1464 TGATTTTCCCTCTAATGGCATAGGCTTTGGTGAATCTTTCAGATTCCAAAAGGTCTTGCA
MtARF4a     1509 TGTTCATCCTTTCAGTTATGCAGGCTTTATGGAAAGTAACAACTTTCCAAGGGTCTTGCA
MtARF4b     1536 GATTCAACCTTCTAGTTATGCAGACTTCACGGAAATGAACCGGTTTCCTAGGGTCTTGCA

MtARF2      1588 ATGCCACCTGGTCTGTCGCTAAATTTCTTGCACTCCA-------------ATATGAAAGG
MtARF3      1524 AGGTCAAGAAATTCTTCTGTGCCCACCCTACGGACGAGCCTCGTTTGA---CGAGGCCCG
MtARF4a     1569 AGGTCAAGAAATATGTAAACTGAAATCCCTTTCAGGAAAAGTTGATTTCAACATTGGAGC
MtARF4b     1596 AGCTCAAGAAATATATCCATTGAGATCCCTGACAGGAAAGGTTGATCTAAACCTTAACTC

MtARF2      1635 TTCT------GCACAAGGCAGTGATAATGCAACTTACCAAGCTCAAGGGAATATGAGATA
MtARF3      1581 TGGAAGTGGTTGCATTGGACGTTATGAT---GGTTATCCAATGCTCGG-CTCCAGAAATG
MtARF4a     1629 TTGGGGAACCAAGCCCAGTTTTCAATCTGCATGTTTTCCT--------------------
MtARF4b     1656 TTGGGGTAAAACCAATGTTAGTTACACAAAATATAACCTGCATAATGC-AACCAAACTTA
```

Supplementary Figure 4. Multiple alignment of nucleotide sequences of the region of *MtARF2, MtARF3, MtARF4a and MtARF4b* transcripts used for the design of *ARF2/3/4* RNAi primers. The multiple alignment was generated the Clustal Omega algorithm available at EMBL-EBI (https://www.ebi.ac.uk/Tools/msa/clustalo/) and shaded with BOXSHADE (http://sourceforge.net/projects/boxshade/). Primers used for amplification of *ARF2/3/4* RNAi fragments are colored in blue. Targets sites for tasiARFs were colored in green. Locus IDs are MtrunA17_Chr8g0385791 (Medtr8g100050), MtrunA17_Chr2g0282961 (Medtr2g014770), MtrunA17_Chr4g0029671 (Medtr4g060460) and MtrunA17_Chr2g0326281 (Medtr2g093740) *MtARF2*, *MtARF3*, *MtARF4a and MtARF4b*, respectively.

Supplementary Figure 5

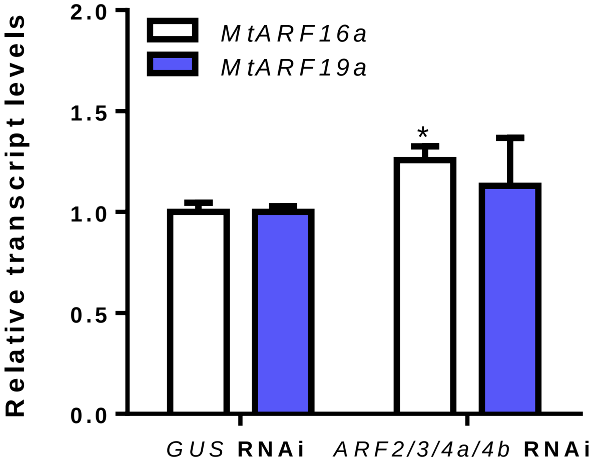

Supplementary Figure 5. Transcript levels of Mt*ARF16a and MtARF19a* in *GUS* RNAi and *ARF2/3/4a/4b* RNAi roots. Expression values were determined by RT-qPCR, normalized to *MtHIS3L* and expressed relative to the *GUS* RNAi sample, which was set at 1. Each bar represents the mean ± SE of three biological replicates (whole root tissue from at least three composite plants were collected in each biological replicate) with three technical replicates each. The asterisk indicates statistically significant differences between *GUS* and *ARF2/3/4a/4b* RNAi roots in an unpaired two-tailed Student's t test with a p value ≤ 0.05.

Supplementary Figure 6

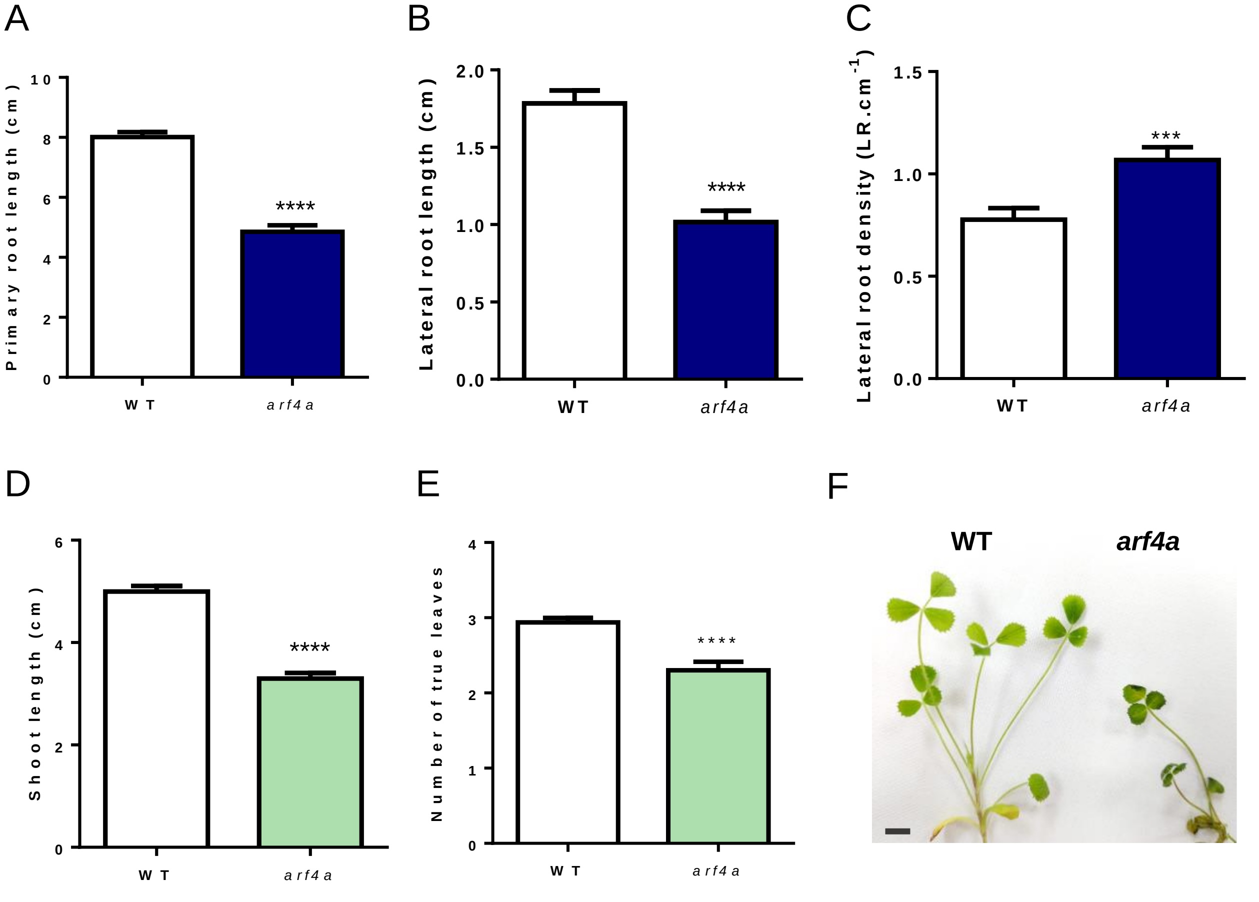

Supplementary Figure 6. Root and shoot phenotype of *arf4a* mutant plants at 15 days after germination (dag). Primary root length (A), lateral root length (B), lateral root density (C), shoot length (D) and number of true leaves (E) were measured in WT and *arf4a* mutant plants at 15 dag. Error bars represent mean ± SE of three biological replicates, each with at least 10 plants. Asterisks denote a statistically significant differences between WT and *arf4a* plants in an unpaired two-tailed Student's t test (**: $p \leq 0.01$, ***: $p \leq 0.001$, ****: $p \leq 0.0001$). F. Illustrative pictures of shoots of WT and *arf4a* plants at 15 dag. Scale bar: 0.5 cm
