# Supplementary material for: Auxin Response Factor 2 (ARF2), ARF3, and ARF4 Mediate Both Lateral Root and Nitrogen Fixing Nodule Development in Medicago truncatula
Source: Front Plant Sci. 2021 Apr 8;12:659061. doi: 10.3389/fpls.2021.659061 (PMC8060633; doi:10.3389/fpls.2021.659061)
Supplement: Supplementary file 2 [file Table_1.DOCX]

**Supplementary Table 1.** **Primers used in this study**

| Primer name | Sequence (5'-3') | Reference |
| --- | --- | --- |
| MtpARF3 F | CACCCCATATCACCCAAACAGAAGCATACC | This work |
| MtpARF3 R | CCTTTTGCAGGAACAGATGAAGCA | This work |
| MtpARF4a F | CACCGGACCAATGTGTTGTAAATGGGTGC | (Hobecker et al., 2017) |
| MtpARF4a R | CAGTGTCGGCTAAGTGGTGAAACT | (Hobecker et al., 2017) |
| *MtARF2/3/4* RNAi F | CACCGGAGCGTCAGACTTTGGGG | This work |
| *MtARF2/3/4* RNAi R | CGTAGGGTGGGCACAGAAG | This work |
| *GUS* RNAi F | CACCGCAACGTCTGGTATCAGCG | This work |
| *GUS* RNAi R | CTGCCAGTTCAGTTCGTTGTTC | This work |
| MtARF2 F | TCGTCAAAAGTAAGCATGGACCC | (Reynoso et al., 2013) |
| MtARF2 R | CCATCTTTTCTTCATCAGTTGCAGGA | (Reynoso et al., 2013) |
| MtARF3 F | CACTTCAGCAAAGCTAGAATTTCCA | (Reynoso et al., 2013) |
| MtARF3 R | TTTGAACCAGGATAGCACCTCCCT | (Reynoso et al., 2013) |
| MtARF4a/b F | AGAAACGGTCTTCCTGAATCAAT | (Reynoso et al., 2013) |
| MtARF4a/b R | TCATTTTGAATCTTGTCCCTATGGT | (Reynoso et al., 2013) |
| MtARF4a F | ACCTTGCATCAACTGGAATAGG | This work |
| MtARF4a R | TTGGTTCCCCAAGCTCCAA | This work |
| MtARF16b F | TTCAAGACATCAAGGAACCTAGCT | This work |
| MtARF16b R | TCATCAAAGGGTTTATTCAGTTGCAAG | This work |
| MtARF19a F | CATCGAGTATTCACATTCTTCGGCA | This work |
| MtARF19a R | ACCATTAAAAGCAGTGTGCGGA | This work |
| MtNSP2 F | GACACACTTGCTGCTTTCCA | (Reynoso et al., 2013) |
| MtNSP2 R | AATGCGGTTATCCGAAGATG | (Reynoso et al., 2013) |
| MtNFY-A1 F | AAAATATGGCTATGCAACCTGTTTA | (Combier et al., 2006) |
| MtNFY-A1 R | CAACTGACATCTTACAATCATCTGG | (Combier et al., 2006) |
| MtNFY-C1 F | CCTGTGATGGACCCAAACA | (Reynoso et al., 2013) |
| MtNFY-C1 R | CACCAATGGAACAGTTTCACC | (Reynoso et al., 2013) |
| MtERN1 F | GGAAGATGGTGCTGTTGCTT | (Andriankaja et al., 2007) |
| MtERN1 R | TGTTGGATTGTGAACCTGACTC | (Andriankaja et al., 2007) |
| MtHISL3 F | ATTCCAAAGGCGGCTGCATA | (Ariel et al., 2010) |
| MtHISL3 R | CTTTGCTTGGTGCTGTTTAGATGG | (Ariel et al., 2010) |

References:

Ariel, F., Diet, A., Verdenaud, M., Gruber, V., Frugier, F., Chan, R., and Crespi, M. (2010). Environmental regulation of lateral root emergence in *Medicago truncatula* requires the HD-Zip I transcription factor HB1. *Plant Cell* 22**,** 2171-2183.

Andriankaja, A., Boisson-Dernier, A., Frances, L., Sauviac, L., Jauneau, A., Barker, D.G., and De Carvalho-Niebel, F. (2007). AP2-ERF transcription factors mediate Nod factor dependent Mt ENOD11 activation in root hairs via a novel cis-regulatory motif. *Plant Cell* 19**,** 2866-2885.

Combier, J.P., Frugier, F., De Billy, F., Boualem, A., El-Yahyaoui, F., Moreau, S., Vernie, T., Ott, T., Gamas, P., Crespi, M., and Niebel, A. (2006). MtHAP2-1 is a key transcriptional regulator of symbiotic nodule development regulated by microRNA169 in Medicago truncatula. *Genes Dev* 20**,** 3084-3088.

Hobecker, K.V., Reynoso, M.A., Bustos-Sanmamed, P., Wen, J., Mysore, K.S., Crespi, M., Blanco, F.A., and Zanetti, M.E. (2017). The MicroRNA390/TAS3 Pathway Mediates Symbiotic Nodulation and Lateral Root Growth. *Plant Physiology* 174**,** 2469-2486.

Reynoso, M.A., Blanco, F.A., Bailey-Serres, J., Crespi, M., and Zanetti, M.E. (2013). Selective recruitment of mRNAs and miRNAs to polyribosomes in response to rhizobia infection in Medicago truncatula. *Plant J* 73 289-301.
